# Phytochromobilin deficiency impairs sugar metabolism through the regulation of cytokinin and auxin signaling in tomato fruits

**DOI:** 10.1038/s41598-017-08448-2

**Published:** 2017-08-10

**Authors:** Ricardo Ernesto Bianchetti, Aline Bertinatto Cruz, Bruna Soares Oliveira, Diego Demarco, Eduardo Purgatto, Lázaro Eustáquio Pereira Peres, Magdalena Rossi, Luciano Freschi

**Affiliations:** 1 Departamento de Botânica, Instituto de Biociências, Universidade de São Paulo, Rua do Matão, 277, 05508-900 São Paulo, Brazil; 2 Departamento de Alimentos e Nutrição Experimental, Faculdade de Ciências Farmacêuticas, Universidade de São Paulo, Av. Professor Lineu Prestes, 580, 05508-000 São Paulo, Brazil; 3 Departamento de Ciências Biológicas, Escola Superior de Agricultura “Luiz de Queiroz”, Universidade de São Paulo, Av. Pádua Dias, 11, CP 09, 13418-900 Piracicaba, Brazil

## Abstract

Phytochomes and plant hormones have been emerging as important regulators of fleshy fruit biology and quality traits; however, the relevance of phytochrome-hormonal signaling crosstalk in controlling fruit development and metabolism remains elusive. Here, we show that the deficiency in phytochrome chromophore phytochromobilin (PΦB) biosynthesis inhibits sugar accumulation in tomato (*Solanum lycopersicum*) fruits by transcriptionally downregulating sink- and starch biosynthesis-related enzymes, such as cell-wall invertases, sucrose transporters and ADP-glucose pyrophosphorylases. PΦB deficiency was also shown to repress fruit chloroplast biogenesis, which implicates more limited production of photoassimilates via fruit photosynthesis. Genetic and physiological data revealed the involvement of auxins and cytokinins in mediating the negative impact of PΦB deficiency on fruit sink strength and chloroplast formation. PΦB deficiency was shown to transcriptionally repress type-A TOMATO RESPONSE REGULATORs and AUXIN RESPONSE FACTORs both in pericarp and columella, suggesting active phytochrome-hormonal signaling crosstalk in these tissues. Data also revealed that PΦB deficiency influences fruit ripening by delaying the climacteric rise in ethylene production and signaling. Altogether, the data uncover the impact of phytochromobilin deficiency in fine-tuning sugar metabolism, chloroplast formation and the timing of fruit ripening and also reveal a link between auxins, cytokinins and phytochromes in regulating sugar import and accumulation in fruits.

## Introduction

Photoreceptor-mediated light perception and signaling have long been described to regulate the development and metabolism of fleshy fruits, ultimately affecting their final composition^1, 2^. In tomato (*Solanum lycopersicum*), the model species for fleshy fruit physiology^3^, red/far-red light perception via phytochromes has emerged as a relevant source of environmental information controlling fruit ripening and carotenogenesis^4–6^. Phytochromes (PHYs) are part of a multigene family, which in tomato encompasses five members: *SlPHYA, SlPHYB1, SlPHYB2, SlPHYE* and *SlPHYF* ^7^. Functional PHYs are homodimers associated with a conserved chromophore known as phytochromobilin (PΦB), which upon red light exposure undergoes a conformational alteration that triggers light signaling^8^. Hence, tomato chromophore-deficient mutants, such as *yellow-green-2* (*yg-2*) and *aurea* (*au*), are deficient in functional phytochromes, resulting in phenotypic changes such as increased stem and petiole elongation as well as pale-green leaves and fruits^3^.

Once activated by light, phytochromes are transported to the nucleus^8^, where they inhibit the PHYTOCHROME INTERACTION FACTORs (PIFs), negative regulators of light response, and promote photomorphogenesis-promoting factors such as LONG HYPOCOTYL 5 (HY5) by downregulating the protein complexes formed by CONSTITUTIVE PHOTOMORPHOGENESIS 1 (COP1), DETIOLATED1 (DET1), DAMAGE DNA BINDING1 (DDB1) and CULLIN4 (CUL4)^8–11^. In accordance with their key functions in light signal transduction, overexpression or knockout/knockdown of tomato genes encoding PIF, HY5, COP1, DET1, DDB1 or CUL4 have been shown to greatly impact the organoleptic and nutritional composition of tomato fruits^12–16^. Hence, manipulating light signaling has been suggested as a viable strategy for improving the nutritional composition of tomato fruits^13, 17^.

Tomato plants with altered light perception or signal transduction frequently show changes in fruit chloroplast biogenesis and development^13, 15, 17^. As developing fruits contain photosynthetically active chloroplasts^18, 19^, increments in the abundance or in the photosynthetic performance of these organelles in immature stages may contribute to some extent in supplying carbon and energy for the highly demanding processes of fruit growth and maturation^20^. Nevertheless, large fleshy fruits, including the modern tomato varieties, are regarded as essentially photosynthate sinks relying on carbon importing from leaves to complete their developmental program^18, 21, 22^. Therefore, carbohydrate metabolism-related enzymes that significantly contribute to the sink activity in developing tomato fruits, such as cell-wall invertases (LINs), sucrose transport proteins (SUTs) and ADP-glucose pyrophosphorylase (AGPases), play central roles in determining fruit growth rates^23–26^. In line with their key role in fruit growth, many of these enzymes are subjected to transcriptional and posttranscriptional regulation by numerous environmental (*e.g*. light, drought, salinity) and endogenous stimuli (*e.g*. plant hormones, carbon availability)^22, 24^.

In tomato, as in many other fleshy fruits, the accumulation of soluble sugars and organic acids is one of the major determinants of the final organoleptic and nutritional quality of the fruits^22^. Remarkable changes in carbohydrate metabolism are observed during tomato fruit development and ripening, all of them directly influencing the final sugar content of the fruit^27^. During early fruit development, starch synthesis and accumulation predominates in the pericarp, which is the most abundant tissue in a tomato fruit; however, the involvement of other tissues in fruit carbon metabolism is also widely accepted. The columella, for instance, plays a central role in importing sugars as this tissue connects the fruit to the rest of the plant^25^. Therefore, the signaling events controlling sugar import and accumulation are presumably coordinated within the different fruit tissues.

Compelling biochemical, genetic and molecular evidence is now revealing that both sources of carbohydrates in fruits (*i.e*. fruit photosynthesis and carbon importing from the leaves) are modulated by both light and hormonal signals^18, 24, 28^. Among plant hormones, auxins and cytokinins are known to control chloroplast biogenesis and differentiation in vegetative and reproductive tissues^28–31^ and also transcriptionally and post-transcriptionally regulate key proteins related to carbohydrate metabolism and sink activity^24, 28, 32^. Despite the massive advances in clarifying the intricate phytochrome-hormonal signaling network controlling various developmental responses in vegetative tissues^33, 34^, the phytochrome and hormonal signaling crosstalk responsible for controlling sugar metabolism in developing fruits remain elusive. Also, limited information is currently available on the role of phytochrome-hormonal interactions in controlling the initiation and progression of fruit ripening. Here, we provide evidence that deficiency in phytochrome chromophore biosynthesis impacts plastid biogenesis and sugar metabolism via coordinated alterations in auxin and cytokinin signaling in both pericarp and columella tissues. The upregulation of genes encoding phytochrome apoproteins in the columella was also identified as a compensatory mechanism to overcome the presumably less R-enriched light reaching fruit inner tissues. Moreover, our data also suggest that PΦB deficiency influences the timing of ripening by delaying the climacteric rise in ethylene production and signaling.

## Results

### PΦB deficiency impacts tomato fruit ripening

Previous studies have shown that mutations in specific phytochromes can significantly alter tomato fruit ripening^4^. Here, the impact of the deficiency in PΦB biosynthesis, which impairs the formation of functional phytochromes, on tomato fruit ripening was determined by monitoring fruit color, carotenoid content as well as ethylene biosynthesis and metabolism during the on-the-vine ripening of fruits from wild type (WT) and PΦB-deficient mutant *aurea* (*au*).

**Figure 1:**
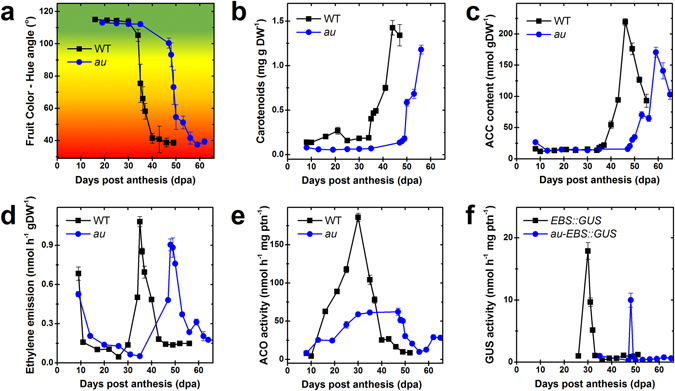
Deficiency in phytochromobilin biosynthesis delays tomato fruit ripening. Ripening-associated traits and ethylene metabolism and signaling were monitored in developing and ripening fruits of wild-type (WT) and *aurea* (*au*) mutant plants. **(a)** Fruit color estimated by Hue angle. **(b)** Total carotenoids content. **(c)** 1-aminocyclopropane-1-carboxylic acid (ACC) content. **(d)** Ethylene emission. **(e)** ACC oxidase (ACO) activity. **(f)** *In vitro* GUS activity assayed in fruits carrying the synthetic ethylene-responsive promoter *EBS* fused to the GUS reporter protein (*EBS::GUS* and *au-EBS::GUS*). Values shown are mean ± SE.

Time course analysis revealed that *au* and WT fruits attained MG stage at virtually the same number of days post anthesis (dpa) and showed no significant differences in fruit diameter throughout the growth and ripening phases (Supplementary Fig. 1A,B). In contrast, the change in fruit coloration from green to yellow, which marks the transition from the MG to the Bk stage, was delayed by approximately 8 days in *au* compared to the WT (Fig. 1A and Supplementary Fig. 1C). After the Bk stage, the progression in the acquisition of distinctive red fruit coloration and the accumulation of carotenoids was indistinguishable in both genotypes (Fig. 1A,B). The delayed-ripening phenotype of *au* fruits ripening on the vine under photoperiodic conditions (Fig. 1) was also observed when fruits of this mutant were harvested at MG and left to ripen off the vine under constant white light or dark conditions (Supplementary Fig. 2). During the off-the-vine ripening, the time required for the transition from the MG to the Bk stage was exactly the same in *au* fruits incubated under either light or dark conditions (Supplementary Fig. 2). Conversely, ripening was initiated earlier in the WT fruits exposed to light than in those maintained under absolute darkness (Supplementary Fig. 2).

In agreement with the changes in fruit coloration, the accumulation of the ethylene precursor 1-aminocyclopropane-1-carboxylic acid (ACC) and the climacteric rise in ethylene emission in *au* fruits were also delayed by approximately 8 days compared to the WT (Fig. 1C,D). Despite such temporal differences, the maximal levels of both ACC content and ethylene emission were only slightly lower in *au* fruits than in the WT (Fig. 1C,D and Supplementary Fig. 3 A,B). In contrast, the activity of ACC oxidase (ACO), a key enzyme in ethylene production^35^, was both delayed and significantly reduced in *au* compared to WT (Fig. 1E). This reduction in ACO activity in *au* was particularly evident in pericarp and columella tissues (Supplementary Fig. 3 C). In both WT and *au* fruits, the climacteric rise in ethylene production coincided with increases in ACO activity and preceded ACC accumulation (Fig. 1C–E). The rise in ACC levels after the climacteric peak of ethylene production suggests that most ACC formed during the climacteric phase is converted into ethylene and the accumulation of the ethylene precursor at the post-climacteric phase may be a consequence of the progressive reduction in ACO activity^36^.

The impact of PΦB deficiency on ethylene signaling was evaluated by measuring the activity of the reporter protein GUS under the control of the ethylene-responsive promoter *EBS* ^37^ and also by analyzing the transcript abundance of tomato genes encoding three ethylene responsive factors (ERFs) – *Sl-ERF.E1*, *Sl-ERF.E2* and *Sl-ERF.E4* – whose expression patterns are closely associated with the ripening process^38^. The peak in ethylene signaling output took place approximately 8 days later in *au* fruits compared to WT, temporally coinciding with the climacteric rise in the production of this hormone in each of these genotypes (Fig. 1F). In both genotypes, the maximum activation of *EBS* promoter was observed earlier in pericarp and columella (Bk1) than in the placental tissues and seeds (Bk12) (Supplementary Fig. 4). In both genotypes, transcript abundance of *Sl-ERF.E2* and *Sl-ERF.E4* progressively increased during ripening (Supplementary Fig. 4). Overall, no marked differences in *Sl-ERF.E1*, *Sl-ERF.E2* and *Sl-ERF.E4* transcript abundance were observed between *au* and WT at each fruit development stage (Supplementary Fig. 4).

Therefore, our data suggest that the PΦB deficiency significantly delays the initiation of the ripening in tomato but has a very limited impact on the progression of ripening once started. Consequently, from this point forward, stage-based comparisons between WT and *au* genotypes will be presented rather than time-course comparisons.

### PΦB deficiency reduces plastid abundance in pericarp cells

**Figure 2:**
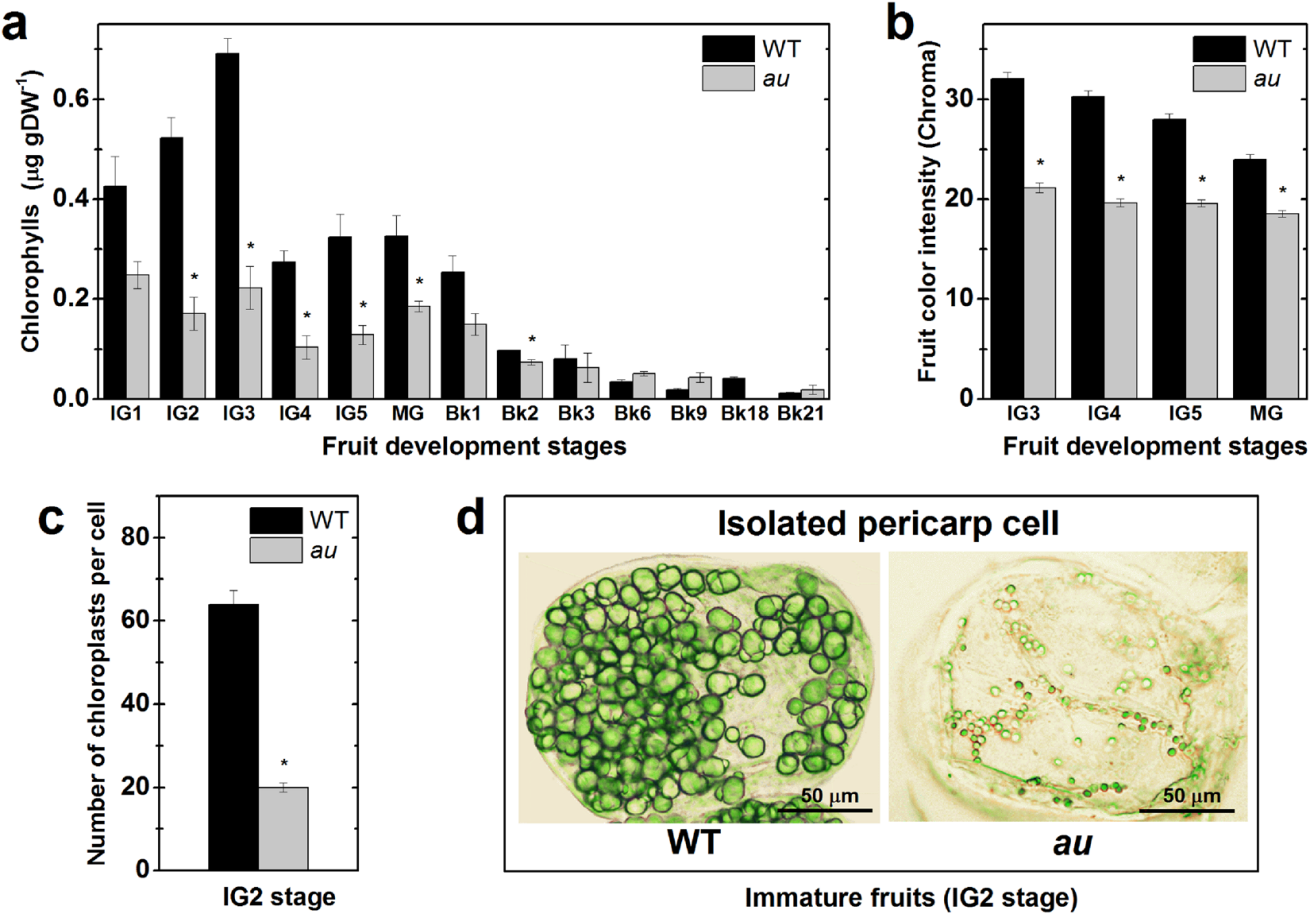
Deficiency in phytochromobilin leads to impaired plastid biogenesis and reduced chlorophyll in pericarp cells. Chlorophyll content, fruit color intensity and plastid abundance per cell were monitored in developing and ripening fruits of wild-type (WT) and *aurea* (*au*) mutant plants. **(a)** Pericarp chlorophyll content. **(b)** Chroma, which indicates color intensity. **(c)** Plastid density per pericarp cell. Values shown are mean ± SE. Asterisks indicate statistically significant differences (Student’s t-test, P < 0.05) compared with the WT at each fruit developmental stage. **(d)** Light microscopy of isolated pericarp cells of immature fruits. Increased chloroplast size and abundance can be observed in WT compared to *au* cells. IG1 to IG5, immature green stages (corresponding to fruit development); MG, mature green; Bk to Bk21, 0 to 21 days after breaker stage (corresponding to the ripening phase).

Among many other processes, phytochromes are known to regulate the accumulation of photosynthetic pigments^39^. Consistently, immature fruits of the *au* mutant exhibited a distinctive pale-green coloration as confirmed both by the reduced total chlorophyll content and significantly lower color saturation (chroma, which is indicative of color intensity) compared to WT (Fig. 2A,B).

As phytochrome signaling can influence both plastid biogenesis and differentiation^39, 40^, it became relevant to assess whether the pale-green phenotype and the associated reduced chlorophyll content in *au* fruits resulted from changes in plastid abundance or alterations in plastid ultrastructural features. Microscopy analysis of pericarp cells revealed a reduction of approximately 70% in the number of chloroplasts per cell in *au* compared to WT (Fig. 2C). Chloroplast were not only less abundant but also smaller in diameter in *au* than in WT (Fig. 2D).

The internal membranous structure of the plastids was remarkably similar in pericarp cells of *au* and WT immature fruits (Supplementary Fig. 5). Well-developed grana and stroma thylakoids were equally observed in both genotypes. In WT fruits, the dismantling of grana thylakoids, indicative of the conversion of chloroplasts into chromoplasts, became evident from the Bk stage onwards. In contrast, dismantlement of grana thylakoids in plastids of *au* pericarp cells was initiated earlier, at the MG stage (Supplementary Fig. 5). During pre-climacteric (IG to MG) and climacteric phases (Bk to Bk3), plastids from *au* pericarp cells exhibited a tendency to present more numerous plastoglobuli than the WT (Supplementary Fig. 5), presumably associated with the accumulation of phytol molecules not diverted to chlorophyll production in this pale-green mutant^41^.

Despite the lower abundance and size of the chloroplasts in unripe fruits of *au* compared to the WT (Fig. 2D), the total carotenoid content was virtually indistinguishable in fully ripe fruits of both genotypes (Fig. 1 and Supplementary Fig. 2). In agreement, chromoplast number per pericarp cell in fully ripe *au* fruits (317.5 ± 71.1) was statistically similar to that observed in WT counterparts (351.8 ± 75.7). These data suggest that intensified plastid replication may occur during the ripening of *au* fruits allowing chromoplasts to accumulate to levels as high as those observed in the WT. This assumption is supported by previous observations in other tomato mutants that also possess extremely reduced chloroplast abundance in unripe fruits but exhibits chromoplast densities similar to the WT at fully ripe stage^42^. Moreover, as plastoglobuli provide the plastids with high capacity to capture and accumulate carotenoids, it seems plausible to suggest that the increased abundance of plastoglobuli in *au* compared to the WT (Supplementary Fig. 5) may also compensate for any differences in chromoplast abundance or size between these genotypes.

Collectively, these data suggest that the distinctive pale-green phenotype and associated reduced chlorophyll content observed in *au* mutant primarily result from a significant reduction in chloroplast abundance and size at pre-climacteric fruit development rather than changes in chloroplast ultrastructure. Data also suggest that intensified plastid replication during the climacteric phase compensate for the limited plastid biogenesis detected in unripe fruits of the PΦB-deficient mutant.

### PΦB deficiency leads to reduced sugar import and accumulation in tomato fruits

**Figure 3:**
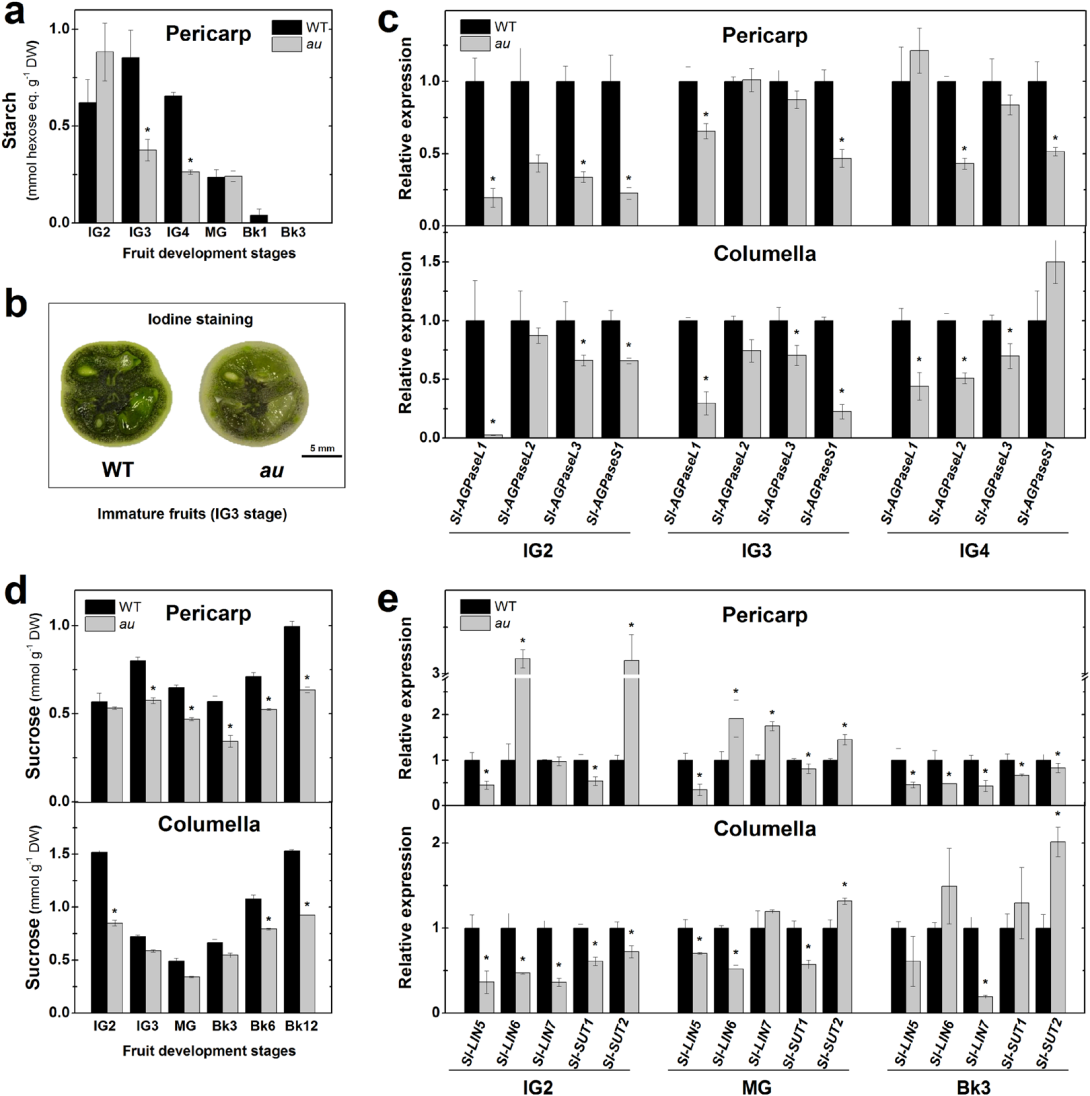
Deficiency in phytochromobilin negatively impacts fruit sugar accumulation and transcript abundance of sink- and starch biosynthesis-related genes. Carbohydrate content and transcript levels of sink-related genes were monitored in developing and ripening fruits of wild-type (WT) and *aurea* (*au*) mutant plants. (**a**) Starch content in pericarp cells. (**b**) Lugol staining in immature fruits (IG3). The blue-purple color indicates starch reaction with iodine. (**c**) Transcript abundance of tomato genes encoding ADP-glucose pyrophosphorylase (*AGPase*) in immature green (IG2 to IG4) fruits. (**d**) Sucrose content in pericarp and columella tissues. (**d**) Transcript abundance of tomato genes encoding invertases (*Sl-LIN*) and sucrose transporters (*Sl-SUT*). Values shown are mean ± SE. Transcript abundance was normalized against WT at each fruit developmental stage. Asterisks indicate statistically significant differences (Student’s t-test, P < 0.05) compared with the WT at each fruit development stage. IG2 to IG4, immature green stages (corresponding to early fruit development); MG, mature green; Bk to Bk12, 0 to 12 days after breaker stage (corresponding to the ripening phase).

It has been estimated that fruit photosynthesis provides up to 20% of the photoassimilates accumulated in this organ^18, 43, 44^. Thus, we further addressed whether the reduced chlorophyll content and plastid abundance are associated with alterations in sugar content in *au* fruits. Indeed, at the onset of fruit expansion, which corresponds to immature green 3 and 4 (IG3 and IG4) stages, reductions of up to 50% in starch content were observed in the pericarp of *au* fruits compared to WT (Fig. 3A). Iodine-staining analysis revealed conspicuously more intense blue-purple color, indicative of the presence of starch, in WT than *au* immature green fruits (Fig. 3B). As expected, starch levels progressively decreased towards undetectable levels at the ripening phase (Fig. 3A). As ADP-glucose pyrophosphorylase (AGPase) represents a limiting enzyme in starch production in tomato fruit^45^, the mRNA levels of genes encoding AGPase large (*Sl-AGPaseL1*, *Sl-AGPaseL2* and *Sl-AGPaseL3*) and small (*Sl-AGPaseS1*) subunits were profiled in immature green fruits (IG2 to IG4). Overall, the mRNA levels of AGPase-encoding genes were significantly reduced in pericarp and columella tissues of *au* compared to the WT (Fig. 3C).

Sucrose and glucose were also significantly less abundant in *au* than in WT during early stages of fruit development (Fig. 3D and Supplementary Fig. 6). At the end of the ripening phase (Bk12), columella and pericarp sucrose levels were approximately 40% lower in *au* than in WT fruits (Fig. 3D). When sucrose, glucose and fructose levels were combined at each fruit development stage, an overall tendency of reduced soluble sugar content in *au* through development was revealed (Supplementary Fig. 6).

Although fruit photosynthesis may provide part of the carbohydrates accumulated in tomato fruits^18, 43, 44^, this organ relies heavily on carbon and energy import from leaves to complete its growth and development^18, 21, 22^. Therefore, to investigate whether the PΦB deficiency also modulates sink-related genes in tomato fruits, a transcriptional profile of genes encoding cell-wall invertases (*Sl-LIN5*, *Sl-LIN6* and *Sl-LIN7*) and sucrose transport proteins (*Sl-SUT1* and *Sl-SUT2*) essential for fruit sink activity^46, 47^ was performed in pericarp and columella samples from both *au* and WT genotypes. In line with the prevailing idea that columella plays a vital role in importing photoassimilates, mRNA levels of *Sl-LIN* and *Sl-SUT* genes were more abundant in this region than in the pericarp (Supplementary Fig. 7). Moreover, consistent with the high demand of carbon and energy required for initial fruit growth, the highest transcript abundances of *Sl-LIN5*, *Sl-LIN6*, *Sl-LIN7* and *Sl-SUT2* in both genotypes were observed during early fruit development and were found to decrease during the ripening phase (Supplementary Fig. 7). Overall, a trend of reduced mRNA levels of *LIN* and *SUT* genes in *au* compared to WT was observed in both columella and pericarp tissues (Fig. 3E). At early fruit development (IG2 stage), all genes were downregulated in the columella of *au* compared to WT fruits (Fig. 3E). Similarly, all *LIN* and *SUT* genes analyzed were also significantly downregulated in pericarp tissues of *au* during ripening (Bk3). Interestingly, *Sl-LIN5*, which encodes the most relevant cell-wall invertase to determine sink activity and brix content in developing tomato fruits^23, 48^, was significantly downregulated in *au* at all fruit development stages and tissues analyzed (Fig. 3E).

The analysis of sugar metabolism showed that PΦB deficiency reduces starch biosynthesis and sink strength, which ultimately leads to decreased carbohydrate levels in fully ripe tomato fruits.

### PΦB deficiency negatively impacts cytokinin and auxin signaling during early fruit development

Among plant hormones, cytokinins and auxins play significant roles in plastid biogenesis and development^28, 29, 49^ as well as sugar import, metabolism and accumulation in tomato fruits^24, 28^. To gain insight into the potential role played by these phytohormones during the phytochrome-dependent regulation of chloroplast development and sugar metabolism in tomato fruits, a spatiotemporal analysis of important cytokinin- and auxin-related signaling components was performed in developing fruits of *au* and WT tomato plants.

**Figure 4:**
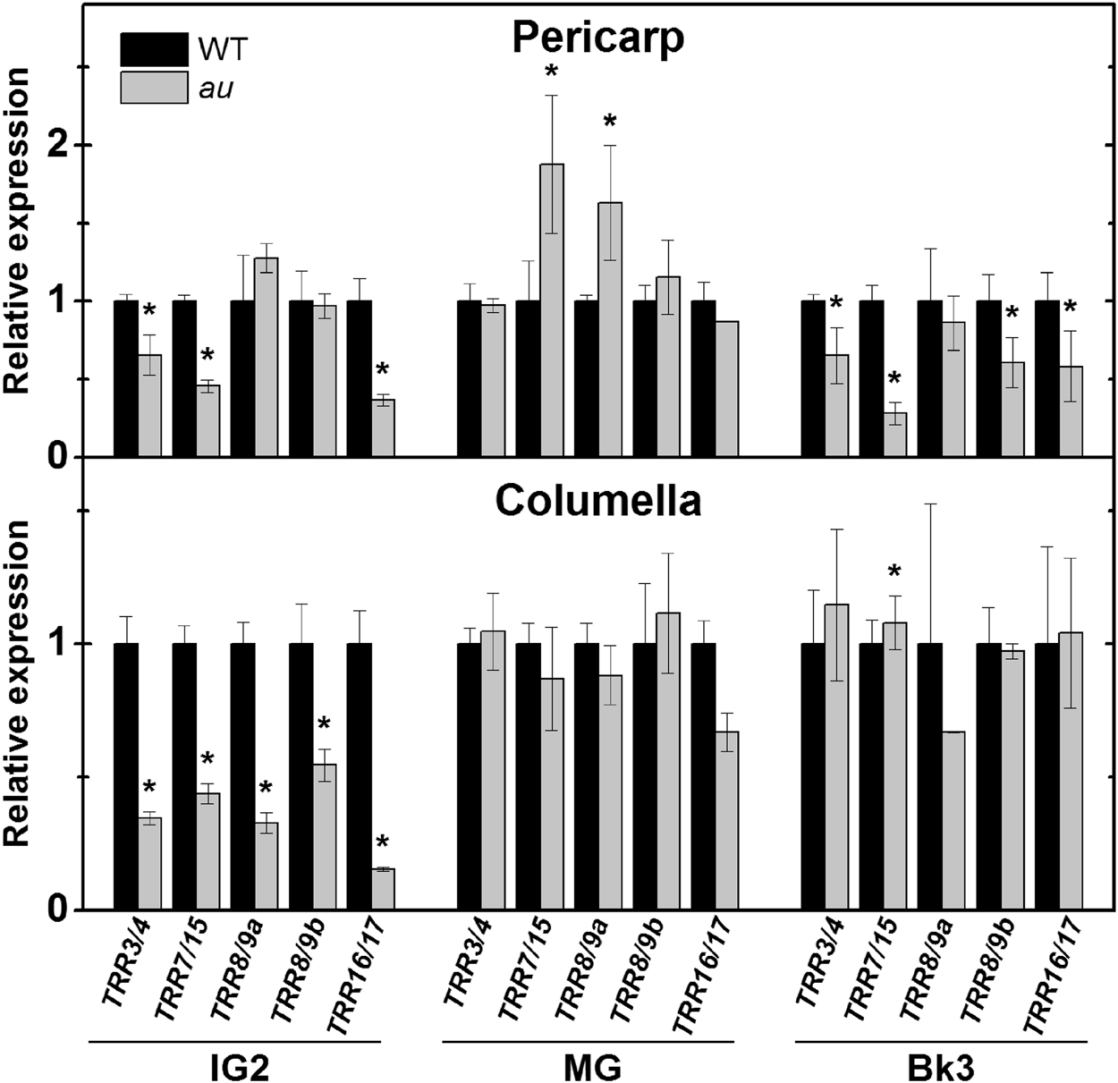
Deficiency in phytochromobilin downregulates primary cytokinin response genes during early tomato fruit development. Transcriptional profile of *TOMATO RESPONSE REGULATOR* (*TRR*) genes was performed in pericarp and columella tissues of immature (IG), mature green (MG) and ripening (Bk3, 3 days after breaker) fruits of wild-type (WT) and *aurea* (*au*) mutant plants. Transcript abundance was normalized against WT at each fruit developmental stage. Asterisks indicate statistically significant differences (Student’s t-test, P < 0.05) compared with the WT at each fruit development stage.

Among the downstream targets of CKs receptors, type-A ARABIDOPSIS RESPONSE REGULATORs (ARRs) represent an important convergence point between phytochrome and cytokinin signaling in vegetative tissues of Arabidopsis^50, 51^. All five type-A *AtARR* homologous in tomato, namely *TOMATO RESPONSE REGULATOR* (*TRR*) *3/4*, *TRR7/15*, *TRR8/9a*, *TRR8/9b* and *TRR16/17* ^52^, were significantly downregulated in the columella of early developing *au* fruits (IG2 stage) compared to the WT (Fig. 4). As observed for *LIN* and *SUT* genes, mRNA levels of most type-A *TRR* genes were higher in columella of early developing fruits, decreasing during the ripening phase (Supplementary Fig. 8).

**Figure 5:**
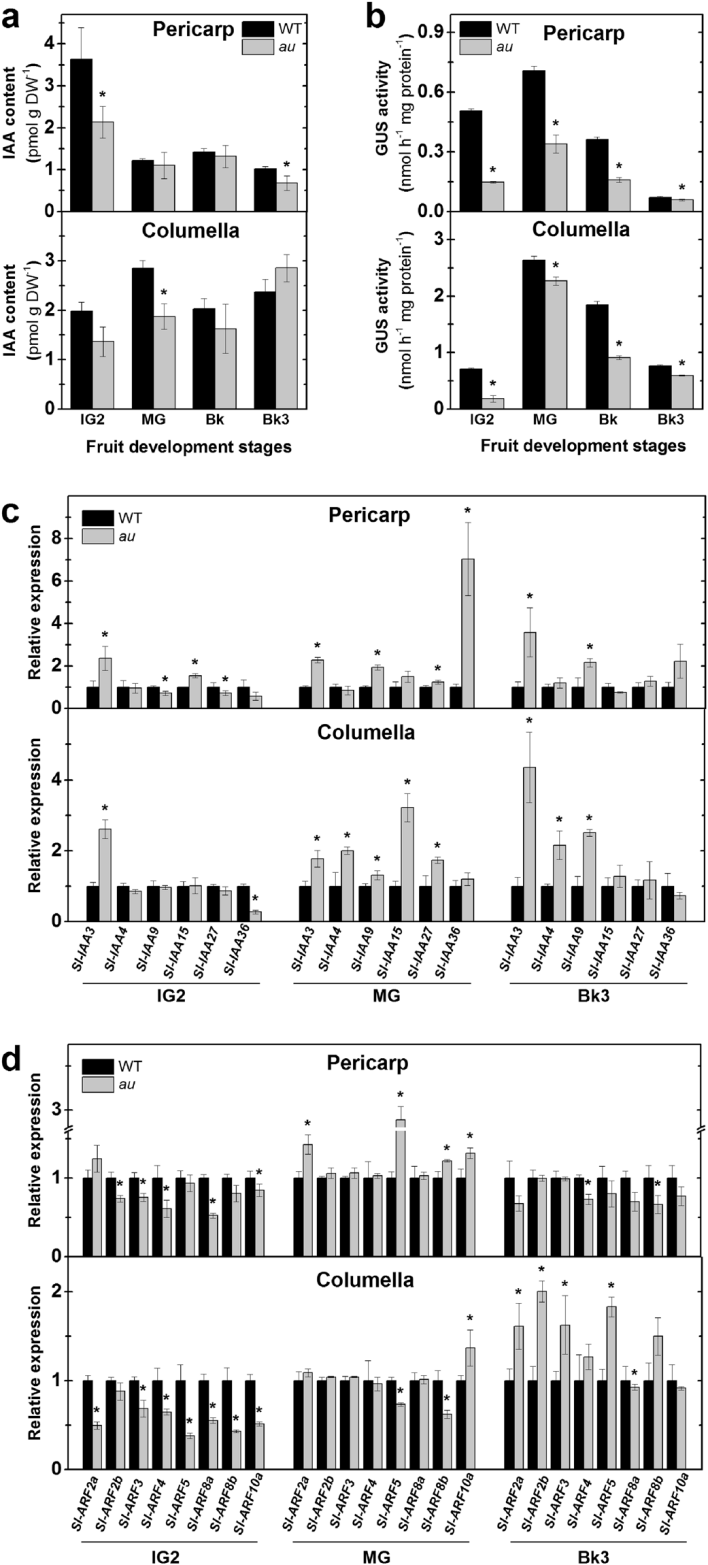
Phytochromobilin deficiency negatively impacts auxin levels and signaling during early tomato fruit development. Auxin content, signaling output and transcript abundance of genes associated with auxin signal transduction were monitored in pericarp and columella tissues of immature (IG), mature green (MG), breaker (Bk) and ripening (Bk3, 3 days after breaker) fruits of wild-type (WT) and *aurea* (*au*) mutant plants. **(a)** Indole-3-acetic acid (IAA) content. **(b)** *In vitro* GUS activity assayed in fruits carrying the synthetic auxin-responsive promoter *DR5* fused to the GUS reporter protein (*DR5::GUS* and *au-DR5::GUS*). **(c)** Relative transcript abundance of *AUXIN/INDOLE-3-ACETIC ACID* (*Aux/IAA*) genes. **(d)** Relative transcript abundance of *AUXIN RESPONSE FACTOR* (*ARF*) genes. The mean relative expression was calculated from means of two technical replicates of at least three biological replicates and normalized against WT at each fruit developmental stage. Values shown are mean ± SE. Asterisks indicate statistically significant differences (Student’s t-test, P < 0.05) compared with the WT at each fruit development stage.

The impact of PΦB deficiency on auxins was first investigated by determining the endogenous content of indole-3-acetic acid (IAA), the main auxin in plants, and the activity of the reporter protein GUS expressed under the control of the *DR5* auxin-responsive promoter in fruits of *DR5::GUS* and *au-DR5::GUS* plants^53^. Whereas *au* and WT fruits exhibited limited differences in IAA content (Fig. 5A), *DR5* promoter activation was significantly lower in *au-DR5::GUS* than in *DR5::GUS* in both columella and pericarp (Fig. 5B). Consistent with the idea that auxin signaling promotes tomato fruit set, cell division and enlargement at pre-climacteric phase while subsequently repressing fruit ripening^54–58^, analysis of the *DR5* promoter activation revealed that, regardless of the genotype or fruit tissue, the maximum auxin signaling output occurs at immature and mature green stages, progressively decreasing during the ripening phase (Supplementary Fig. 9). Histochemical GUS staining revealed that the auxin-responsive *DR5* promoter is more intensively active in the inner pericarp and columella tissues (Supplementary Fig. 10), which is consistent with the higher *in vitro* GUS activity detected in the columella of *DR5::GUS* and *au-DR5::GUS* compared to other fruit tissues (Supplementary Fig. 9).

Given the marked differences in *DR5* promoter activation between *au* and WT developing fruits, a transcriptional profile of genes encoding auxin-associated signaling proteins was performed. In the auxin signaling cascade, hormone perception leads to targeting Aux/IAA (AUXIN/INDOLE-3-ACETIC ACID INDUCIBLE) proteins for degradation via 26S proteasome, thus reducing the cellular abundance of these key repressors of auxin response^28, 59^. Among the 26 members of the tomato *Aux/IAA* family, *Sl-IAA3, Sl-IAA4, Sl-IAA9, Sl-IAA15, Sl-IAA27* and *Sl-IAA36* have been identified as the most prominently expressed in fruit tissues^59^; consequently, their transcript abundance was compared in developing *au* and WT fruits. At the MG stage, mRNA levels of most *Sl-IAA* genes in both columella and pericarp were significantly higher in *au* than WT (Fig. 5C). Compared to WT, *Sl-IAA3* was upregulated in both columella and pericarp tissues of *au* in all fruit development stages analyzed. Except for *Sl-IAA3*, the expression of all *IAA* genes analyzed was significantly downregulated at ripening (Bk3) in columella and pericarp tissues of both genotypes (Supplementary Fig. 11).

Aux/IAA proteins are known to inhibit the expression of auxin-associated genes by a constant physical inhibition of ARF transcript factors^28, 60^, which in tomato are encoded by a gene family comprising 22 members^60^. Therefore, the transcript abundance of eight *Sl-ARF* genes highly expressed in fruits (*i.e. Sl-ARF2a*, *Sl-ARF2b*, *Sl-ARF3*, *Sl-ARF4, Sl-ARF5*, *Sl-ARF8a*, *Sl-ARF8b* and *Sl-ARF10a*) was also evaluated^28, 61^. Compared to WT, most of the *Sl-ARF* genes analyzed were significantly downregulated in the columella and pericarp tissues of *au* early developing fruits (IG2 stage) (Fig. 5D). With the exception of *Sl-ARF2a* and *Sl-ARF5*, mRNA levels of all other *Sl-ARF* genes were especially high during early fruit development, decreasing thereafter (Supplementary Fig. 12).

The analysis of the transcriptional profile of *TRR*, *Sl-IAA* and *Sl-ARF* genes revealed that the deficiency in PΦB biosynthesis negatively impacts cytokinin and auxin signaling. Moreover, the presence of PBE-box, G-box, CA-hybrid and/or CG-hybrid motifs^62, 63^ within the 3-kb promoter sequence of most of these genes (Supplementary Figs 13 and 14) supports the hypothesis that HY5 and PIF transcription factors may directly control the expression of these cytokinin- and auxin-signalling genes.

### Auxin signaling influences sugar metabolism in tomato fruit

The widely accepted positive role played by cytokinins in tomato fruit sink activity and sugar accumulation^24^ contrasts with the considerably more limited information regarding auxin influence on sugar import and accumulation during fruit development^28^. To further characterize the impacts of auxin signaling on tomato fruit sugar metabolism, the starch content, as well as the transcript levels of sink- and starch biosynthesis-related genes were determined in a *Sl-ARF4*-silenced line (*SlARF4-ASL*)^28^ and in the *Sl-IAA9* loss-of-function mutant *entire* ^64^.

**Figure 6:**
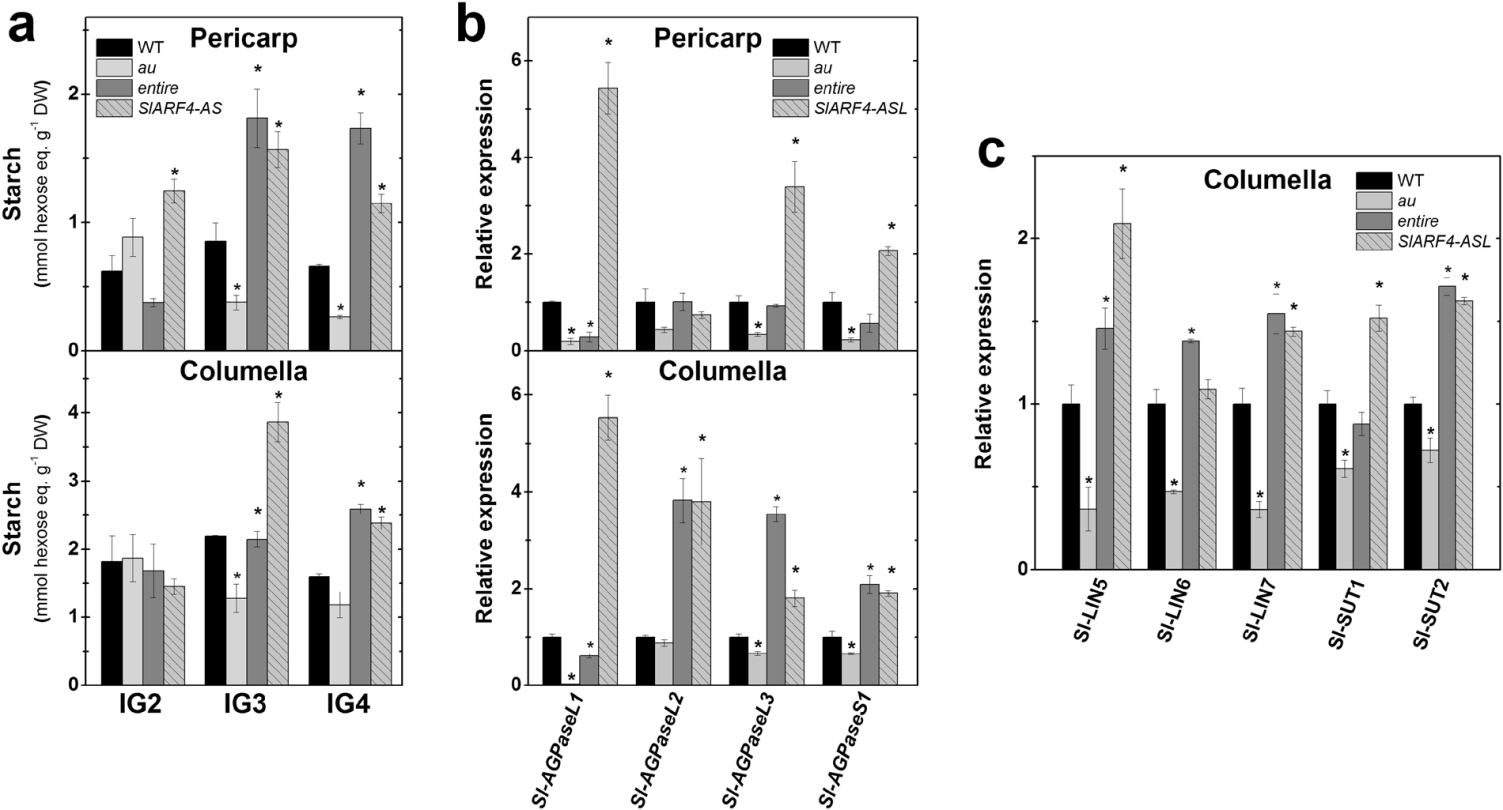
Auxin promotes starch accumulation by increasing transcript abundance of sink- and starch biosynthesis-related genes. Starch content and transcript levels of sink- and starch biosynthesis-related genes were monitored during the early development of *aurea* (*au*), *entire* and *Sl-ARF4*-silenced line (*SlARF4-ASL*) fruits. **(a)** Starch content in pericarp and columella tissues of immature green (IG2 to IG4) fruits. **(b)** Transcript abundance of tomato genes encoding ADP-glucose pyrophosphorylase (*AGPase*) in pericarp and columella tissues of immature green 2 (IG2) fruits. **(c)** Transcript abundance of tomato genes encoding invertases (*Sl-LIN*) and sucrose transporters (*Sl-SUT*) in columella tissues of IG2 fruits. Values shown are mean ± SE. Transcript abundance was normalized against WT at each fruit developmental stage. Asterisks indicate statistically significant differences (Student’s t-test, P < 0.05) compared with the WT. IG2 to IG4, immature green stages (corresponding to early fruit development).

Overall, *Sl-ARF4*-*ASL* and *entire* exhibited significantly higher levels of starch than the WT during most of the initial fruit development (Fig. 6A). These differences were particularly more pronounced at IG3 and IG4 stages, thus coinciding with the fruit development stages in which the starch levels were conspicuously lower in *au* than in the WT (Fig. 6A). Accordingly, *SlAGPase* genes were upregulated in both pericarp and columella samples of immature fruits of the *Sl-ARF4*-*ASL* line (Fig. 6B). Compared to WT, columella mRNA levels of *Sl-AGPaseL2, Sl-AGPaseL3* and *Sl-AGPaseS1* also increased in the *entire* mutant (Fig. 6B). Among the *AGPase* genes, *Sl-AGPaseL2* expression was particularly disturbed in both the *au* mutant and the *Sl-ARF4*-silenced line (Fig. 6B). Interestingly, *Sl-ARF4*-*ASL* and *entire* immature fruits exhibited increased transcript abundance of most sink-related genes analyzed, and the opposite was observed the for the *au* mutant (Fig. 6C). Although the direct transcriptional regulation of tomato *AGPase*, *LIN* and *SUT* genes by auxin or light signaling-associated transcription factors remains to be determined, the presence of canonical and/or degenerated ARF-binding Auxin Response Element (AuxRE) motifs as well as *cis*-acting elements recognized by HY5 and/or PIFs within the 3 Kb fragment promoter of all these genes (Supplementary Fig. 15) is consistent with this hypothesis.

### Phytochrome genes are highly expressed in tomato fruit columella

The most striking differences in transcript abundances of auxin-, cytokinin- and sink-related genes between *au* and WT were observed in the columella. As pericarp and columella are exposed to distinct light intensity and quality conditions^12^, the mRNA levels of phytochromes and light signaling-related genes were profiled.

**Figure 7:**
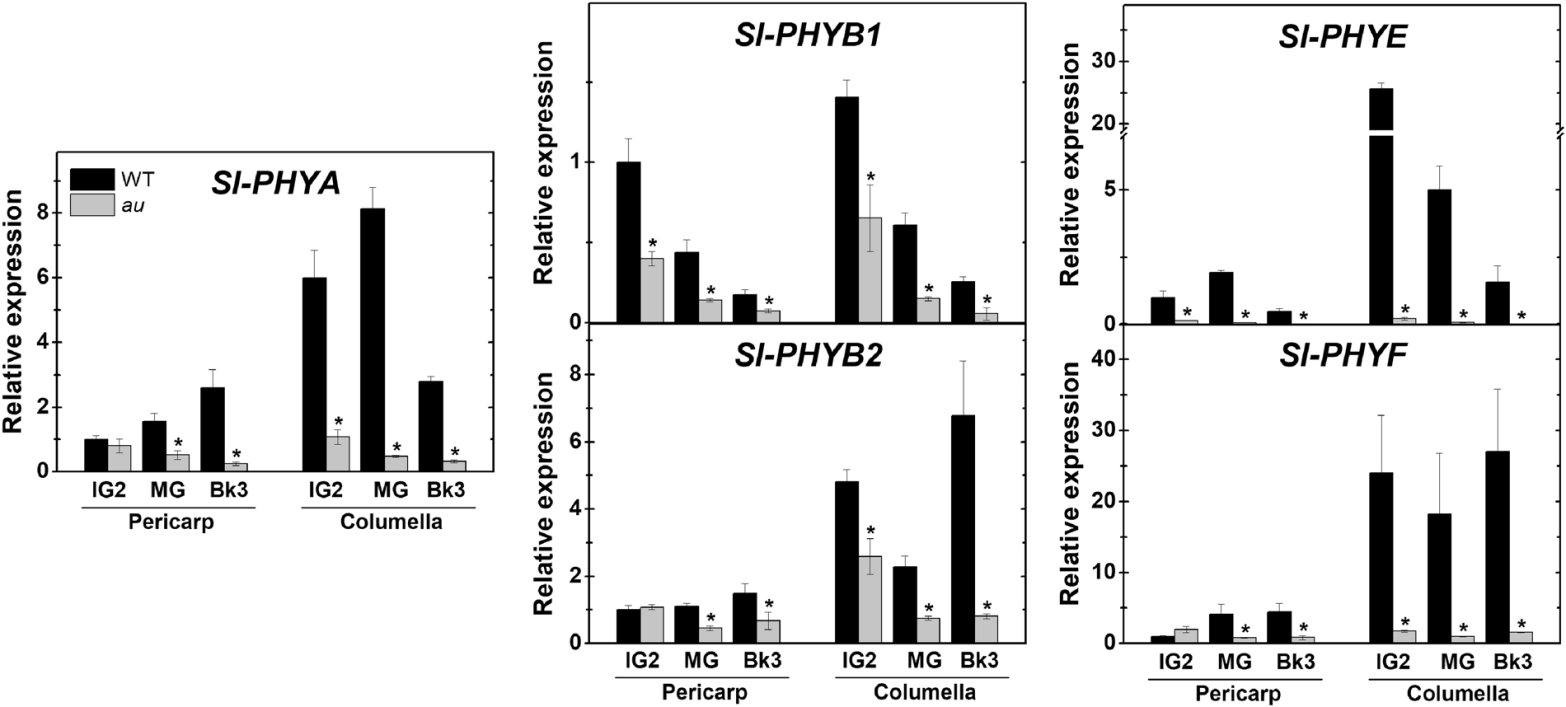
Deficiency in phytochromobilin downregulates PHY-encoding genes. Relative mRNA levels of the five tomato genes encoding phytochromes (*Sl-PHY*) in the pericarp and columella tissues of wild type (WT) and *aurea* (*au*) fruits harvested at immature (IG2), mature green (MG) and ripening (Bk3) stages. Mean relative expression was normalized against pericarp WT samples at IG2 stage. Values shown are mean ± SE. Asterisks indicate statistically significant differences (Student’s t-test, *p* < 0.05) between *au* and WT at each fruit development stage.

Interestingly, the mRNA levels of all five genes encoding phytochromes in tomato were markedly higher in columella than in pericarp tissues (Fig. 7). In early fruit development (IG2), mRNA levels of *Sl-PHYA*, *Sl-PHYB1* and *Sl-PHYB2* were up to four-fold higher in columella than in pericarp, whereas for *Sl-PHYE* and *Sl-PHYF* the differences reached up to 20–30-fold. *Sl-PHYB1* and *Sl-PHYE* mRNA levels progressively reduced during ripening in columella, whereas less noticeable patterns in transcript abundances were observed for the other PHY-encoding genes (Fig. 7). It is worth mentioning that *Sl-PHYB2* mRNA was the PHY-encoding gene most abundantly expressed in all the tissues and stages evaluated (Table S1). Transcript levels of all tomato PHY-encoding genes were drastically reduced in *au* fruits compared to the WT, which may reflect a negative feedback mechanism triggered by the deficiency in PΦB of this mutant (Fig. 7). Regarding the light signaling-associated genes analyzed, *Sl-COP1*, *Sl-DDB1* and *Sl-DET1* showed similar mRNA levels in *au* and WT; however, *Sl-CUL4* transcript abundance was remarkably lower in *au* fruits in both, pericarp and columella (Supplementary Fig. 16). Negligible differences in mRNA levels were observed between columella and pericarp regardless of genotype or fruit development stage analyzed.

Collectively, these data suggest that light perception and signal transduction may be active even in the most internal tissues, and the more intensive expression of *PHY*s might compensate for the reduced amount of incident radiation in columella. Moreover, the data also revealed that all PHY-encoding genes are markedly downregulated in the *au* mutant, which further suggests that this PΦB-deficient mutant may represent a suitable model for investigating the impact of global deficiency of functional phytochromes on tomato fruit development and metabolism.

## Discussion

The role of light and hormones in plastid biogenesis and development as well as in sugar import, metabolism and accumulation in tomato fruits has been assessed in several studies^17, 26, 29, 33^; however, whether and how the signaling cascades triggered by these stimuli crosstalk remains elusive. Here, the impact of the potential interconnection between light and hormone signaling in tomato fruit metabolism was investigated by applying a mutant-based approach and time course analysis of two functionally and spatially contrasting fruit tissues: the pericarp and the columella.

It is widely accepted that climacteric tomato fruit ripening is triggered only after the cell expansion phase is complete and seeds are mature^65^. This dramatic change in the developmental program is marked by an intermittent rise in ethylene production and signaling^65^. Whereas distinct effects on fruit growth and ripening progression were observed in *phyA*, *phyB1*, *phyB2*, *phyAB1*, *phyB1B2* and *phyAB1B2* tomato mutants^4^, here we demonstrate that deficiency in phytochrome chromophore synthesis leads to a significant delay in the initiation of the fruit ripening process. However, no impact was observed on the time course of fruit growth, in the magnitude of climacteric ethylene production and signaling, nor in the progression of ripening once started (Fig. 1). During normal vegetative growth, when auto-inhibitory ethylene production predominates, phytochrome-dependent light perception has been shown to downregulate ethylene biosynthesis^53, 66^. In contrast, here we provide genetic evidence that the deficiency in PΦB biosynthesis postpones the autostimulatory ethylene production in tomato fruit, consequently delaying the initiation of ripening.

Another distinctive phenotypical trait observed in early developing *au* fruits was the lower chloroplast abundance and the reduced levels of chlorophyll conferring their characteristic pale-green phenotype (Fig. 2). These findings have several implications. First, they agree with the increased plastid biogenesis widely reported in loss-of-function or silencing of tomato genes encoding light signaling repressors such as DET1, DDB1, CUL4 and COP1^13–16^. Second, fruit photosynthesis and consequently the local production of photoassimilates might be limited, thus explaining, at least in part, the lower carbohydrate levels observed in this mutant and reinforcing the fact that fruit photosynthesis is a source of carbon for this organ^19, 42, 43^. Third, besides their role in fruit photosynthesis, plastids are also critical for the accumulation of starch granules in immature tomato fruits and are the site of action of AGPases, which are chloroplast-localized enzymes^67^. Therefore, the reduced starch accumulation and lower transcript abundance of *AGPase* genes observed in *au* fruits may simply reflect the limited chloroplast biogenesis observed in this PΦB-deficient mutant. Additionally, the impact of the impaired phytochrome chromophore biosynthesis on sugar metabolism was not only the result of the reduced number of chloroplasts but also the weaker sink strength in *au* fruits evidenced by the downregulation of cell wall invertases and sucrose transporters (Fig. 3).

The simultaneous reduction in transcript abundance of *Sl-ARF* and type-A *TRR* genes, associated with the upregulation of some particular *Sl-IAA* genes (*e.g*., *Sl-IAA3*) and the reduction in *DR5* promoter activity observed in *au* immature fruits (Figs 4 and 5) strongly suggest that phytochrome deficiency negatively influences cytokinin and auxin signaling in tomato fruits. The downregulation of type-A *TRR* genes observed in immature *au* fruits is in line with the key role described for type-A ARRs in red light signaling and in the control of photoresponsive events in vegetative tissues of Arabidopsis^51, 68^, suggesting that similar phytochrome-cytokinin interaction mechanisms might also take place during the early development of tomato fruits. Whether the negative impact of the PΦB deficiency on type-A *TRR* mRNA levels correlates with changes in the endogenous levels of cytokinins, as demonstrated in other plant models^69^, remains to be investigated.

Interestingly, as the reduction in the auxin-responsive *DR5* promoter activity observed in *au* fruits was not accompanied by significant changes in endogenous auxin content, auxin responsiveness rather than biosynthesis or transport seems to be the primary phytochrome-auxin link during the early stages of tomato fruit development. Considering the fact that *Aux/IAA* genes encode short-lived proteins that bind to and repress the ARF transcript factors^60^, one possible explanation for the altered auxin responsiveness observed in the PΦB-deficient mutant is the direct transcriptional regulation of *Aux/IAA* and *ARF* genes by light signaling-associated transcription factors. This is supported by the presence of PIF and/or HY5 binding motifs within the promoters of most of these genes. Moreover, biochemical evidence indicates that at least some Arabidopsis Aux/IAA proteins are post-translationally regulated by phytochrome-mediated phosphorylation^70^; consequently, the direct influence of phytochrome on tomato Aux/IAA protein stability and action is also a promising venue deserving further investigation.

Genetic and pharmacological evidence clearly indicates that cytokinins are key promoters of invertase activity, sink activity and sugar accumulation in developing tomato fruits^24^. It is also known that exogenous auxins can promote both sugar metabolism^71^ and activity of sucrose-cleaving enzymes^24^ in immature tomato fruits. In agreement, our data show that the downregulation of *Sl-ARF4*, which is known to act as a repressor rather than a promoter of the transcription of auxin-responsive genes^28^, results in increased starch content along with higher mRNA levels of most AGPase-encoding genes in early developing tomato fruits (Fig. 6), which agrees with previous reports using this genotype^28^. Importantly, sink-related genes were also upregulated in the *SlARF4*-suppressed line, thus suggesting that not only starch accumulation^28^ but also sugar import may be negatively regulated by this auxin transcriptional regulator. Moreover, the detection of increased starch content and mRNA levels of genes involved in starch synthesis and sink activity in immature fruits of the auxin-constitutive response mutant *entire* (Fig. 6) further supports the positive role of auxin on sugar metabolism in tomato fruits. In agreement with our findings, fruits of an *Aux/IAA9* frame-shift tomato mutant identified via TILLING-based screening exhibited an increased brix value compared to the WT in the fully ripe stage under either greenhouse or open-field conditions^57^. Therefore, although the direct regulation of tomato *AGPase*, *LIN* and *SUT* genes by light signaling-associated transcription factors also cannot be excluded due to the presence of HY5 and/or PIF bindings motifs in their promoter regions, it seems plausible to hypothesize that the reduced sugar content and transcript abundance of sink- and starch biosynthesis-related genes observed in early developing *au* fruits may be associated with the lower cytokinin and auxin signaling output detected in this PΦB-deficient mutant compared to the WT.

**Figure 8:**
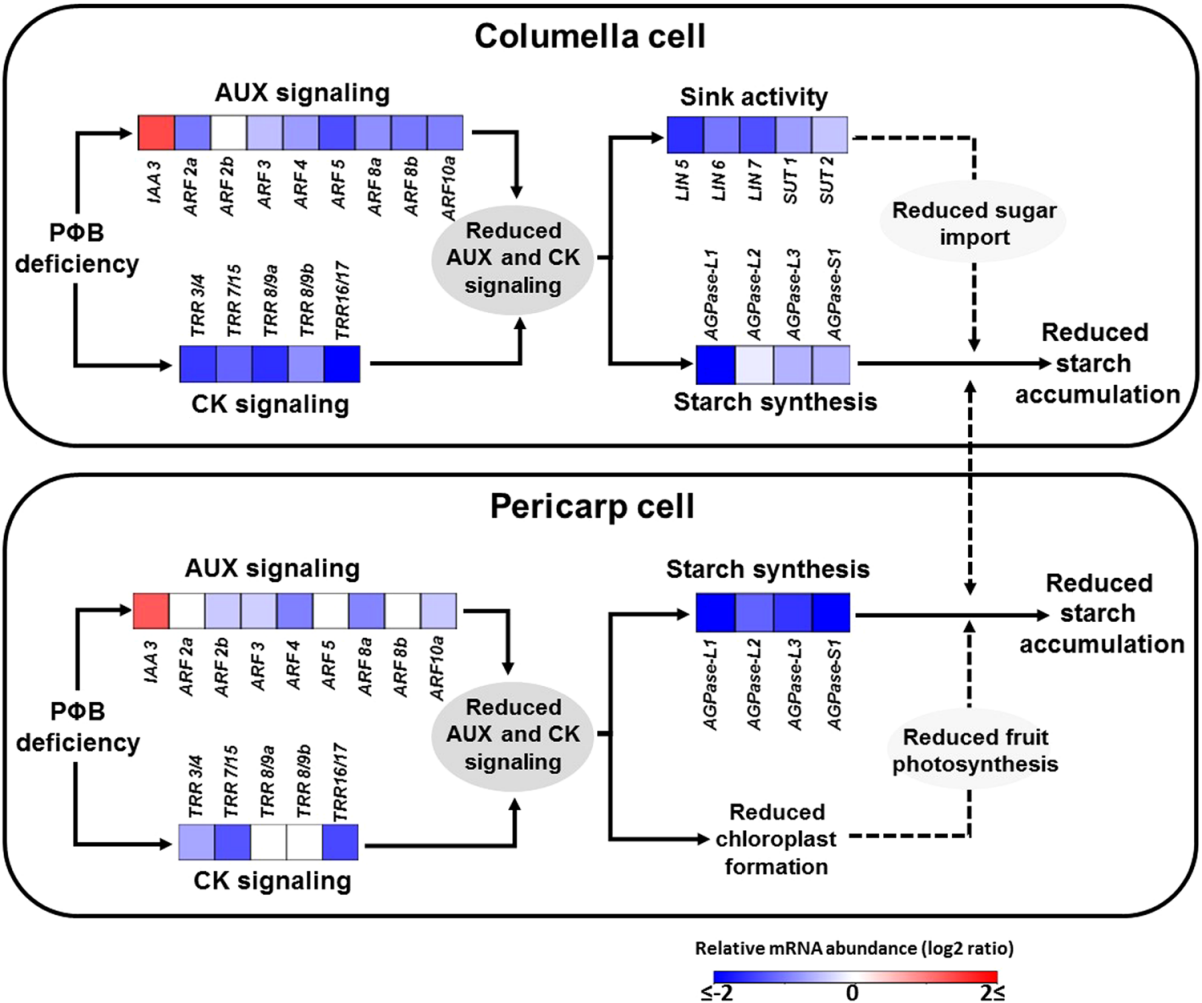
Impact of phytochrome chromophore deficiency on auxin and cytokinin signaling and its potential influence on sugar metabolism and plastid development during early tomato fruit development. At immature green stage, phytochrome chromophore phytochromobilin (PΦB) deficiency upregulates *AUXIN/INDOLE-3-ACETIC ACID3* (*Aux/IAA3*) and downregulates *AUXIN RESPONSE FACTOR* (*ARF*) and type-A *TOMATO RESPONSE REGULATOR* (*TRR*) genes in both columella and pericarp tissues, consequently leading to reduced auxin (AUX) and cytokinin (CK) signaling output in both of these fruit regions. In columella cells, the PΦB-deficiency-triggered reduction in AUX and CK signaling downregulates genes encoding key enzymes involved in starch synthesis, such as ADP-glucose pyrophosphorylase (AGPases), and sink activity, such as cell-wall invertases (LINs) and sucrose transport proteins (SUTs). In pericarp cells, similar PΦB-deficiency-triggered changes in hormonal signaling inhibit both starch biosynthesis and chloroplast formation. The downregulation of sink-related genes in columella is believed to reduce sugar import to the fruit, whereas the impaired chloroplast formation in pericarp cells may restrict local photoassimilate production via fruit-localized photosynthesis. Therefore, the reduced fruit starch accumulation in PΦB-deficient plants may be associated with the more limited sugar import and fruit photosynthesis in this genotype. Arrows at the ends of lines indicate stimulatory influence. Dotted lines indicate hypothesized mechanisms. Transcript abundance data were expressed as the ratio between qPCR values obtained in PΦB-deficient (*aurea*) and wild-type samples at the immature green 2 (IG2) stage, corresponding to early fruit development.

Finally, despite their contrasting localization within tomato fruits, pericarp and columella tissues exhibited surprisingly similar phytochrome-dependent hormonal changes during early fruit development (Fig. 8), which suggests that phytochrome signaling cascade may be active in both of these fruit regions. Recent reports indicate a self-shading effect as sunlight passes through the flesh of green tomato fruits, resulting in a progressive reduction in both light transmittance and R/FR ratios at increasing depths within the fruit tissues^12^. Therefore, internal fruit regions, such as the columella, presumably have a significantly higher proportion of phytochromes in their inactive form compared to more externally positioned fruit tissue layers such as the pericarp. As markedly higher transcript abundance of all tomato phytochrome-encoding genes was observed in tomato columella than in pericarp tissues, we are left to speculate whether potential increments in phytochrome apoprotein abundance in the columella represent a compensatory mechanism to overcome the presumably less R-enriched light reaching the inner tissues of tomato fruit. The limited difference in transcript abundance of genes encoding light signaling-associated proteins, such as COP1, DDB1, DET1 and CUL4, in pericarp and columella tissues is consistent with such a hypothesis.

As the deficiency in PΦB inexorably limits the formation of functional phytochromes, the downregulation of all tomato PHY-encoding genes in *au* suggests that the production of the PHY apoproteins in the plant cells may be inhibited via a negative feedback loop mediated by the accumulation of nonfunctional molecules of this photoreceptor. Therefore, besides the limited abundance of PΦB molecules, the *au* mutation also leads to a global deficiency in PHY apoproteins, thus reinforcing the relevance of this genotype for investigating the impacts of global phytochrome deficiency on fruit biology and quality traits. However, it is plausible to assume that not all phenotypical alterations detected in *au* may be directly associated with its deficiency in functional phytochromes as the impaired production of PΦB molecules may also affect other physiological processes^72^. Hence, additional studies including single and multiple *phy* tomato mutants might be instrumental to further evaluate the phytochrome-dependent regulation of tomato fruit sugar metabolism and plastid development. Moreover, it is also important to highlight that all findings presented in this work were obtained using Micro-Tom, a dwarf tomato variety that contains a weak mutation related to brassinosteroid biosynthesis^73^; therefore, further studies are also needed to determine whether phytochrome-hormonal signaling connections similar to those described here are also observed in “non-dwarf” tomato cultivars.

Altogether, the data showed that PΦB deficiency impacts chloroplast formation and sugar metabolism during early tomato fruit development via changes in both cytokinin and auxin signaling, revealing a link between hormones and phytochromes that regulates sugar import, metabolism and accumulation in tomato fruits. This study also opens up a window of opportunity for improving fruit ripening, sink strength and sugar content through the combined manipulation of phytochrome- and hormone-related genes.

## Material and Methods

### Plant material and growth conditions

Seeds of wild type (WT) *Solanum lycopersicum* L. (cv. Micro-Tom), *aurea* (*au*) and *entire* mutants and transgenic lines carrying the synthetic auxin-responsive (*DR5*) and ethylene-responsive (*EBS*) promoters fused to the reporter gene *uid* (encoding a β-glucuronidase, GUS) were obtained from the Laboratory of Hormonal Control of Plant Development (www.esalq.usp.br/tomato)^3^. The *EBS::GUS* construct was donated by Dr. Anna N. Stepanova and Dr. Jose M. Alonso (North Carolina State University, USA). Seeds of Micro-Tom *SlARF4*-silenced line (*SlARF4*-ASL) were obtained from Dr. Mohamed Zouine and Dr. Mondher Bouzayen (University of Toulouse)^28^. Crosses and phenotypical screening were carried out to generate *au-DR5::GUS* and *au-EBS::GUS*. All the analyzed plants were in the Micro-Tom background and homozygous for the mutations or transgenes.

### Growth conditions and treatments

Plants were grown in 6-L rectangular pots containing a 1:1 mixture of commercial substrate (Plantmax HT, Eucatex, São Paulo, Brazil) and expanded vermiculite, supplemented with 1 g L^−1^ of NPK 10:10:10, 4 g L^−1^ of dolomite limestone (MgCO_3 _ + CaCO_3_) and 2 g L^−1^ thermophosphate (Yoorin Master®, Yoorin Fertilizantes, Brazil) in growth chamber at 250 µmol m^−2^ s^−1^, 12-h photoperiod, air temperature of 27 °C day/22 °C night and air humidity of approximately 60% day/80% night. Immature green (IG) fruits were harvested about 5, 8, 10, 15 and 22 days after anthesis (dpa) corresponding to the stages IG1, IG2, IG3, IG4 and IG5, respectively. Fruits were also harvested after reaching the following ripening stages: MG (ca. 30 dpa, displaying jelly placental tissues), Bk (breaker, ca. 34 dpa, displaying the first external yellow color signals) and Bk1, Bk2, Bk3, Bk6, Bk9, Bk12, Bk15, Bk18 and Bk21, corresponding to 1, 2, 3, 6, 9, 12, 15, 18 and 21 days after breaker, respectively. Fruits were divided into pericarp, columella and placental tissues + seeds. All biological samples were harvested at the same time of the day to avoid possible fluctuations in the parameters due to circadian rhythm. Four biological samples formed by at least five fruits each were harvested at each sampling time. Ethylene emission, iodine-staining and quantitative GUS analyses were performed immediately after harvesting. For other assays, fruits tissues were frozen in liquid nitrogen, ground and stored at −80 °C.

### Pigment quantification and fruit color

Chlorophyll extraction and quantification were carried out as described in Lira *et al*.^74^. Carotenoids were extracted and quantified as described in Sestari *et al*.^75^. Change in Hue angle and fruit color intensity (chroma) during tomato fruit development and ripening were measured using a Konica Minolta CR-400 colorimeter^56^.

### Starch and soluble sugar quantification

Soluble sugars and starch were extracted as described in Lira *et al*.^74^. The supernatant residue was measured in a HPLC system equipped with a pulsed amperometric detector (Dionex, Sunnyale, USA) and a CarboPac PA1 (4 × 250 mm) column as described in Purgatto *et al*.^76^. The endogenous metabolite concentration was obtained by comparing the peak areas of the chromatograms with commercial standards. Starch content was determined from dried pellets as described in Suguiyama *et al*.^77^.

### Hormonal analysis

Endogenous indoleacetic acid (IAA) levels were determined by gas chromatography tandem mass spectrometry-selected ion monitoring (GC-MS-SIM). Frozen samples (100 mg FW) were extracted and methylated as described in Rigui *et al*.^78^. Approximately 0.5 μg of labeled [^13^C_6_]IAA (Cambridge Isotopes, Inc.) was added to each sample as an internal standard. Extracts were analized on a gas chromatograph (GC) coupled to a mass spectrometer (MS) (model GCMS-QP2010 SE, Shimadzu) in selected ion monitoring mode as described in Melo *et al*.^53^. Ions with a mass ratio/charge (m/z) of 130 and 189 (corresponding to endogenous IAA) and 136 and 195 (corresponding to [^13^C_6_]-IAA) were monitored. Endogenous concentrations were calculated based on extracted chromatograms at m/z 130 and 136.

Ethylene emission was determined in intact tomato fruits detached and enclosed in a sealed transparent vial. The vial was flushed with ethylene-free air (1 L min^–1^) for 5 min and incubated for 60 minutes under specific experimental conditions, as appropriate. After incubation, 1-mL gas samples were analyzed as described in Melo *et al*.^53^.

Endogenous ACC was extracted and analyzed as described by Bulens *et al*.^35^. Activity of ACO was determined according to Bulens *et al*.^35^, with the modifications described in Melo *et al*.^53^.

### Hormone-responsive promoter analysis

Quantitative GUS activity assay was assayed according to Jefferson *et al*.^79^, with some modifications. Briefly, samples were ground in liquid nitrogen and subsequently homogenized in MUG extraction buffer composed of 50 mM Hepes-KOH (pH 7.0), 5 mM DTT and 0.5% (w/v) PVP. After centrifugation, 200 µL aliquots of the supernatant was mixed with 200 µL GUS assay buffer composed of 50 mM HEPES-KOH (pH 7.0), 5 mM DTT, 10 mM EDTA and 2 mM 4-methylumbelliferyl-β-D-glucuronide (MUG) and incubated at 37 °C for 30 minutes. Subsequently, aliquots of 100 µL were taken from each tube and the reactions were stopped and fluorescence was analyzed using a spectrofluorometer (LS55, Perkin Elmer) with 365 nm excitation and 460 nm emission wavelength (5 nm bandwidth).

### Plastid ultrastructure and abundance

Pericarp samples were cut into small pieces (1 × 1 mm) and plastid ultrastructure was analyzed as described in Melo *et al*.^53^.

Plastid abundance was determined as in Li *et al*.^80^ with some modifications. Briefly, small pericarp pieces (1 × 1 mm) were incubated in glutaraldehyde 3.5% (v/v) for 60 min and cells were separated by incubation in Na-EDTA 0.1 M pH 9.5 at 60 °C for 180 min under continuous agitation. Isolated cells were visualized using a Leica microscope, and plastid densities were estimated on individual cells using the ImageJ program.

### Transcriptional profile

Total RNA extraction, cDNA synthesis, primer design and qPCR assays were performed as described by Quadrana *et al*.^81^. Primer sequences used are detailed in Supplementary Table S2. qRT-PCR reactions were performed in a StepOnePlus PCR Real-Time thermocycler (Applied Biosystems) in a final volume of 10 µl using 2X SYBR Green Master Mix reagent (Thermo Fisher Scientific). Melting curves were checked for unspecific amplifications and primer dimerization. Absolute fluorescence data were analyzed using the LinRegPCR software package to obtain quantitation cycle (Cq) values and calculate primer efficiency. Transcript abundances were normalized against the geometric mean of two reference genes, *CAC* and *EXPRESSED* ^82^.

### Gene promoter analyses

Promoter sequences were retrieved from Sol Genomics Network (https://solgenomics.net/) and analyzed using PlantPAN 2.0 platform (http://plantpan2.itps.ncku.edu.tw/ ^83^) to identify the regulatory motifs. Typical 3 kb upstream from the initial codon ATG was analyzed for the presence of PBE-box (CACATG), G-box (CACGTG), CA-hybrid (GACGTA) and CG-hybrid (GACGTG) motifs, which are recognized by HY5 and/or PIFs^62, 63^, as well as the presence of canonical AuxRE (TGTGTC) and degenerate AuxRE (TGTGNC), which are recognized by ARFs^84^.

## Electronic supplementary material

Supplementary Information
