# Supplementary material for: Phytochromobilin deficiency impairs sugar metabolism through the regulation of cytokinin and auxin signaling in tomato fruits
Source: Sci Rep. 2017 Aug 10;7:7822. doi: 10.1038/s41598-017-08448-2 (PMC5552807; doi:10.1038/s41598-017-08448-2)
Supplement: Supplementary file 1 — Supplementary Information [file 41598_2017_8448_MOESM1_ESM.pdf]

Phytochromobilin deficiency impairs sugar metabolism through the regulation of cytokinin and auxin signaling in tomato fruits

Authors: Ricardo Ernesto Bianchetti, Aline Bertinatto Cruz, Bruna Soares Oliveira, Diego Demarco, Eduardo Purgatto, Lázaro Eustáquio Pereira Peres, Magdalena Rossi, Luciano Freschi

The following Supplementary Information is available for this article:

Supplementary Figure 1. Comparative fruit development and ripening in wild type and phytochromobilin-deficient mutant.

Supplementary Figure 2. Impact of light treatment on tomato fruit ripening.

Supplementary Figure 3. Ethylene metabolism in developing tomato fruits.

Supplementary Figure 4. Ethylene signaling in developing tomato fruits.

Supplementary Figure 5. Transmission electron microscopy of plastids from pericarp cells.

Supplementary Figure 6. Soluble carbohydrate profile in developing tomato fruits.

Supplementary Figure 7. Transcript abundance of sink-related genes in tomato fruits.

Supplementary Figure 8. Transcript abundance of *TRR* genes in tomato fruits.

Supplementary Figure 9. Auxin signaling output in developing tomato fruits.

Supplementary Figure 10. Spatial pattern of *DR5::GUS* expression during fruit development.

Supplementary Figure 11. Transcript abundance of *Aux/IAA* genes in tomato fruits.

Supplementary Figure 12. Transcript abundance of *ARF* genes in developing tomato fruits.

Supplementary Figure 13. HY5- and PIF-binding motifs identified in the promoter region of *TRR* genes.

Supplementary Figure 14. HY5- and PIF-binding motifs identified in the promoter region of *Aux/IAA* and *ARF* tomato genes.

Supplementary Figure 15. HY5-, PIF- and ARF-binding motifs identified in the promoter region of sink- and starch biosynthesis-related tomato genes.

Supplementary Figure 16. Transcript abundance of light signaling-related genes in developing tomato fruits.

Table S1. Relative transcript ratio of *Sl-PHY* genes in pericarp and columella tissues.

Table S2. Primer sequences used for qPCR.

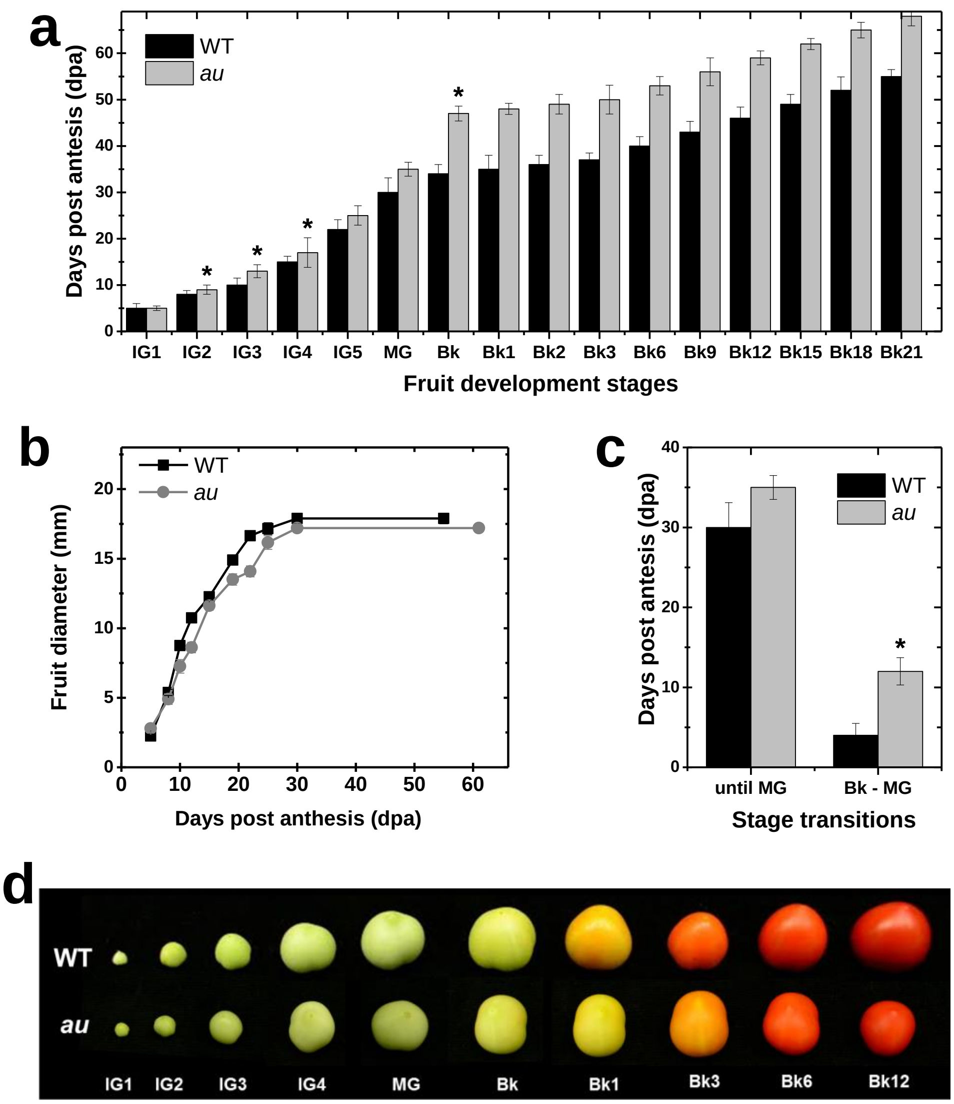

Supplementary Figure 1. Comparative fruit development and ripening in wild type and phytochromobilin-deficient mutant. Chronological development and time course analysis of increase in diameter of wild-type (WT) and *aurea* (*au*) fruits harvested at five immature green stages (IG1 to IG5), mature green (MG), Bk (breaker), Bk1 (1 day after Bk), Bk2, Bk3, Bk6, BK9, Bk12, Bk15, Bk18 and Bk21. **(a)** Time interval in days post anthesis (dpa) to attain each of these fruit sampling stages. **(b)** Time course of increase in fruit diameter from anthesis until fully ripe stage. Diameter was recorded using a digital caliper and the time interval to attain specific development stages was monitored in on-the-vine ripening fruits ($n \geq 20$). **(c)** Time interval (dpa) from anthesis to MG and from MG to Bk stage. **(d)** Representative image of WT (above) and *au* (below) fruits at distinct development stages. Asterisks indicate statistically significant differences compared with the WT at each fruit development stage (Student's t-test, $P<0.05$).

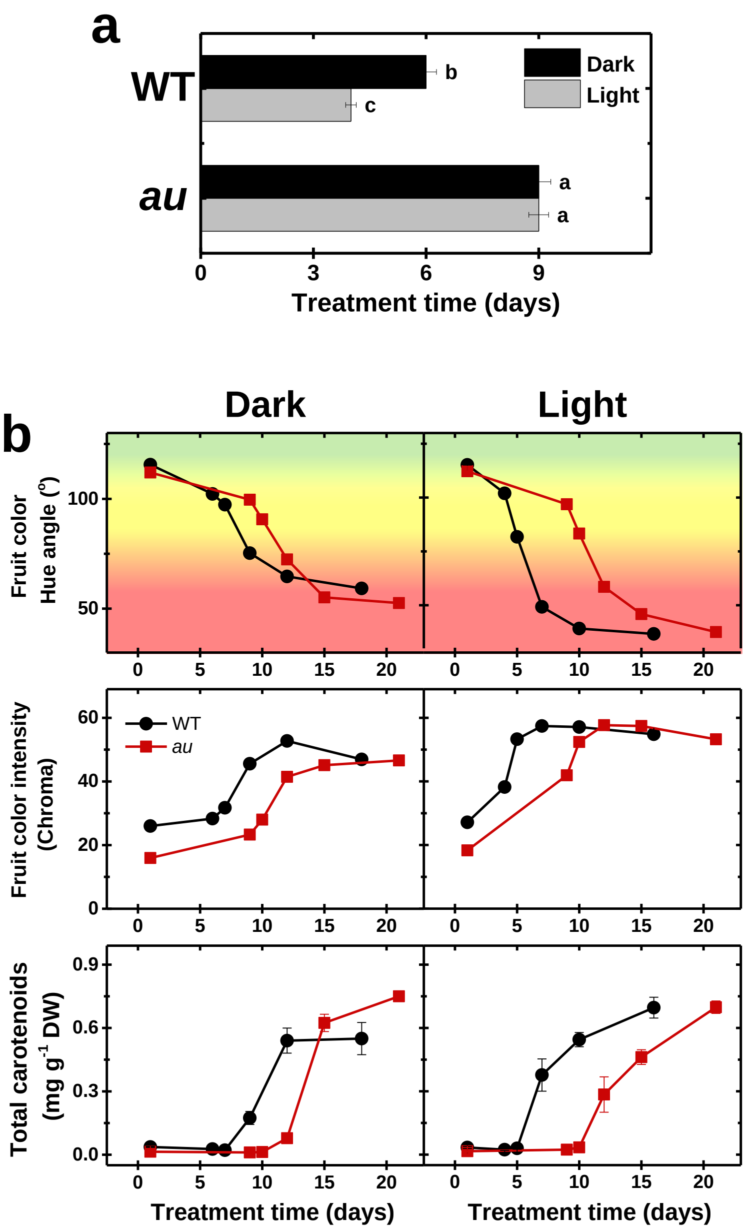

Supplementary Figure 2. **Impact of light treatment on tomato fruit ripening.** Fruits of wild-type (WT) and *aurea* (*au*) mutant were left to ripen off-the-vine under constant white light (approximately 50 µmol m^{-2} s^{-1}) or absolute dark conditions. Samples were harvested at mature green (MG, two days after the beginning of the treatment), breaker (Bk), Bk1 (1 day after Bk), Bk3, Bk6 and Bk12 stages. (**a**) Time required for the transition from MG to Bk stage. Different letters indicate statistically significant differences (Tukey's test, $p < 0.05$) within genotypes and light treatments. (**b**) Ripening-related changes in fruit color (Hue angle), fruit color intensity (Chroma) and total carotenoid content. Values shown are mean ± SE.

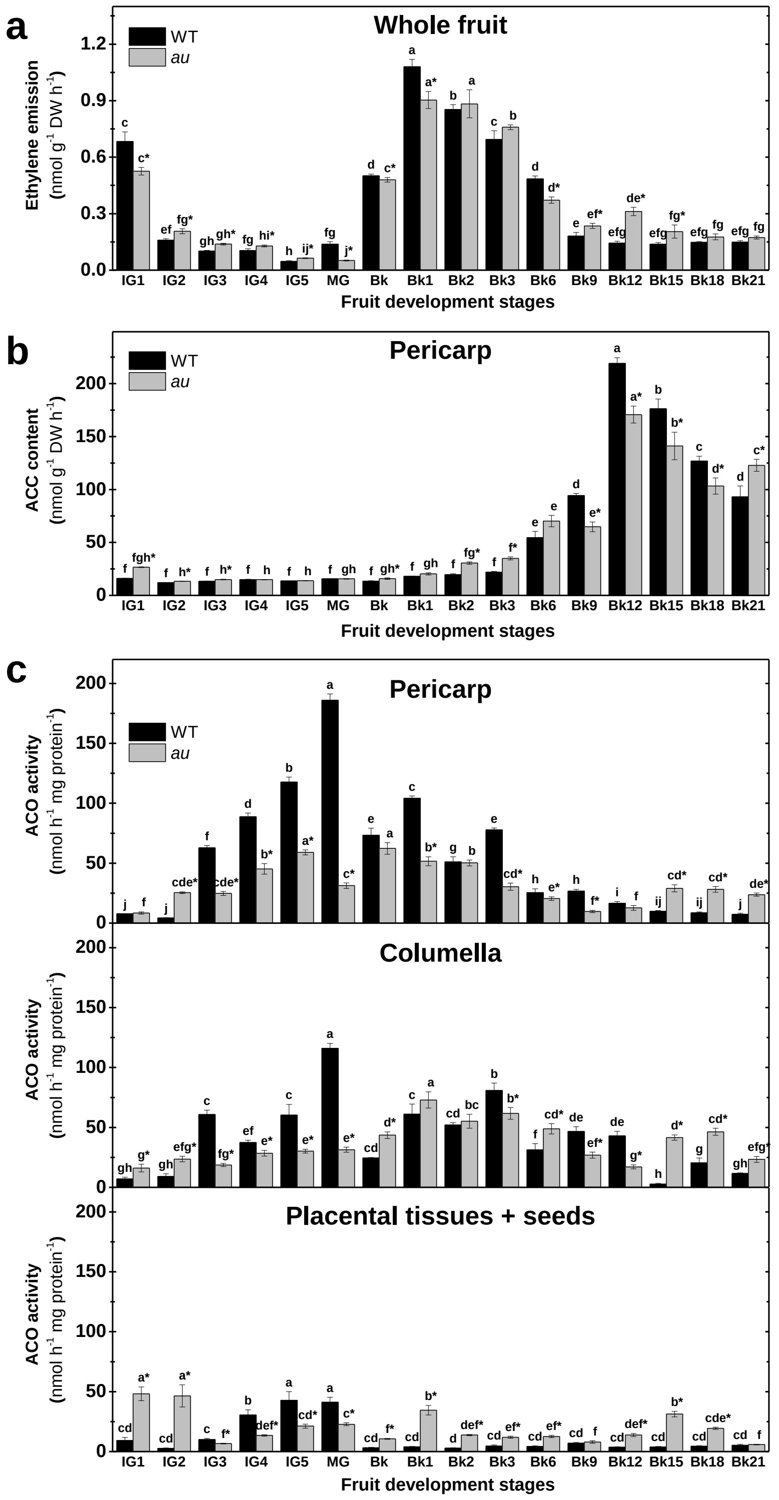

Supplementary Figure 3. Ethylene metabolism in developing tomato fruits. Comparison of ethylene metabolism in wild-type (WT) and *aurea* (*au*) fruits harvested at five immature green stages (IG1 to IG5), mature green (MG), Bk (breaker), Bk1 (1 day after Bk), Bk2, Bk3, Bk6, BK9, Bk12, Bk15, Bk18 and Bk21. **(a)** Ethylene emission in whole fruits. **(b)** 1-aminocyclopropane-1-carboxylic acid (ACC) content in pericarp tissues. **(c)** ACC oxidase

(ACO) activity in the pericarp, columella and placental tissues + seeds. Values shown are mean ± SE. Different letters indicate statistically significant differences (Tukey's test, $p < 0.05$) among fruit development stages within each genotype. Asterisks indicate statistically significant differences (Student's t-test, $p < 0.05$) between *au* and WT at each fruit development stage.

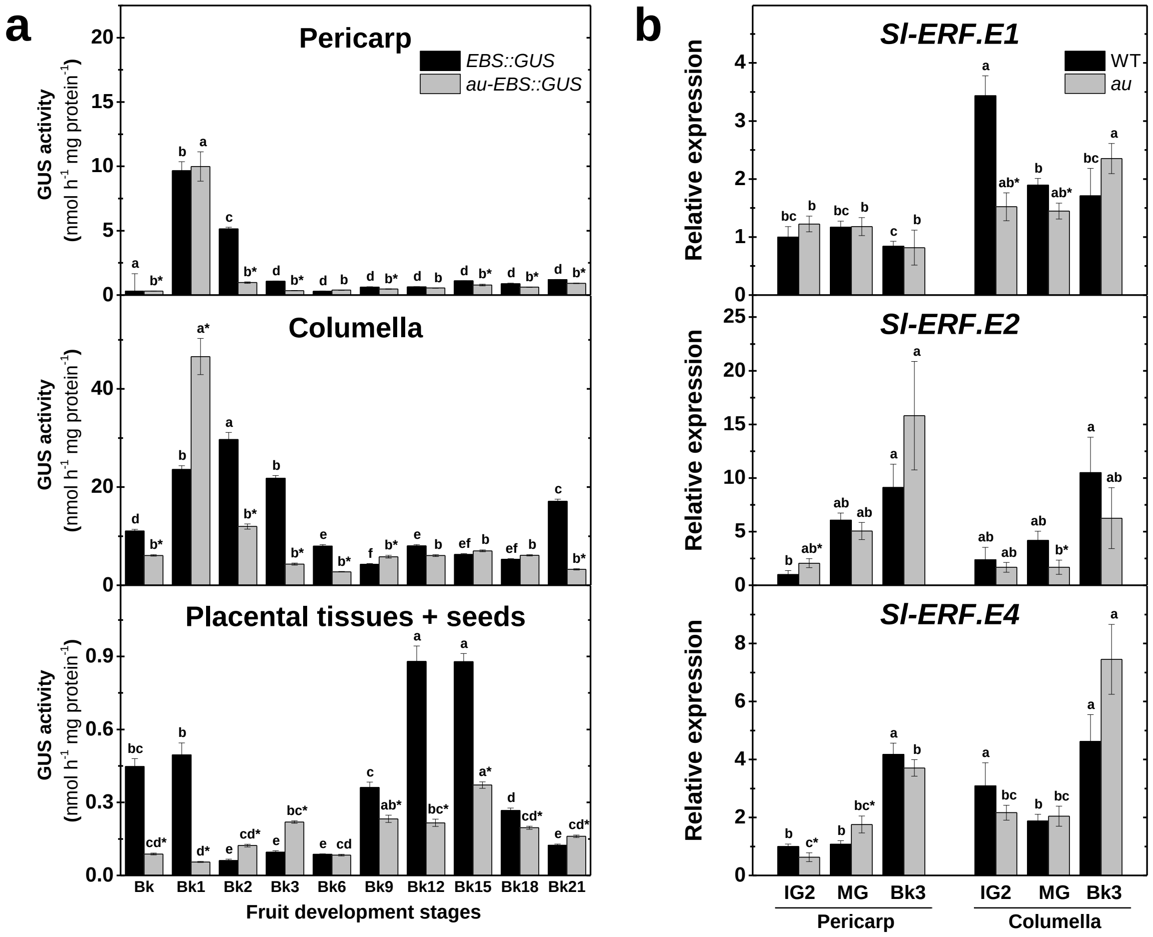

Supplementary Figure 4. Ethylene signaling in developing tomato fruits. Comparison of ethylene signaling output in developing wild-type (WT) and *aurea* (*au*) fruits. **(a)** *In vitro* GUS activity assayed in the pericarp, columella and placental tissues + seeds of ripening fruits carrying the ethylene-responsive promoter *EBS* fused to the GUS reporter protein (*EBS::GUS* and *au-EBS::GUS*). **(b)** Transcript abundance of genes encoding the ethylene-responsive factors (ERFs) *Sl-ERF.E1*, *Sl-ERF.E2* and *Sl-ERF.E4* in the pericarp and columella tissues of immature (IG2), mature green (MG) and ripening (Bk3) fruits. Mean relative expression was normalized against pericarp WT samples at IG2 stage. Values shown are mean ± SE. Different letters indicate statistically significant differences (Tukey's test, $p < 0.05$) among fruit development stages within each genotype. Asterisks indicate statistically significant differences (Student's t-test, $p < 0.05$) between *au* and WT at each fruit development stage.

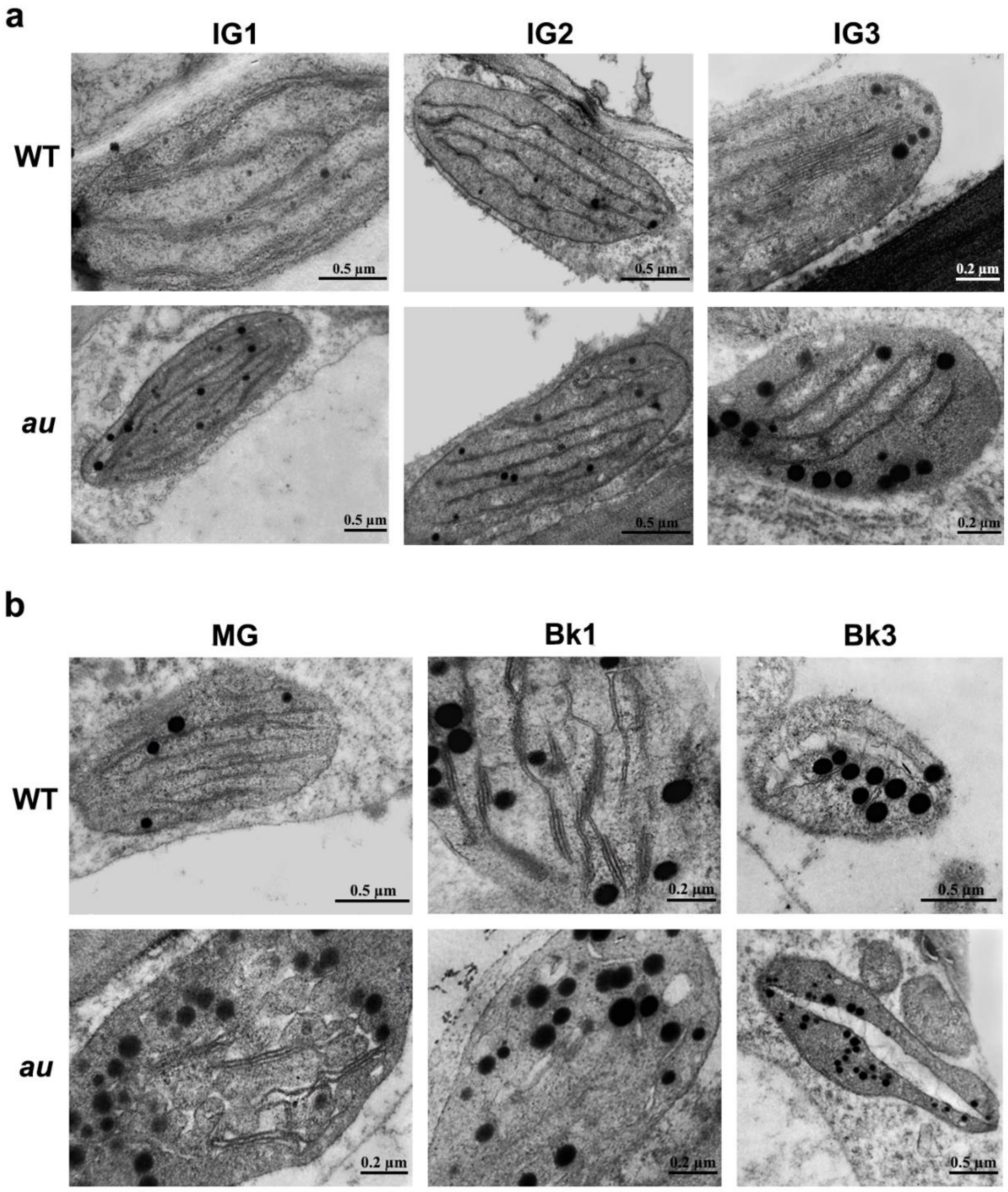

Supplementary Figure 5. Transmission electron microscopy of plastids from pericarp cells. (a) Immature green fruits (IG1 to IG3). **(b)** Mature green (MG) and ripening fruits (Bk1 and Bk3, 1 and 3 days after breaker stage, respectively). Typical thylakoids including grana stacks can be observed in both wild-type (WT) and *aurea* (*au*) immature green fruits. Precocious chloroplast-to-chromoplast conversion and accumulation of plastoglobuli can also be observed in *au* mutant at MG stage.

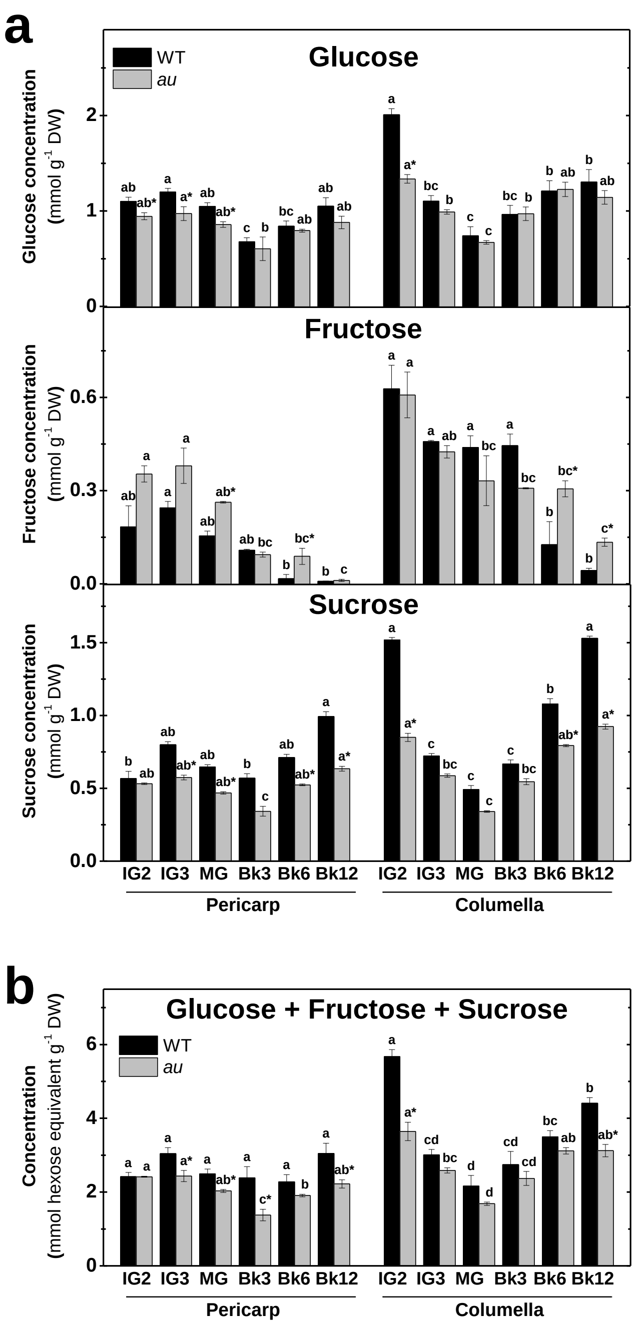

Supplementary Figure 6. Soluble carbohydrate profile in developing tomato fruits. Comparison of carbohydrate profile in wild-type (WT) and *aurea* (*au*) fruits harvested at two immature green stages (IG2 and IG3), mature green (MG), Bk3 (3 day after Breaker), Bk6 and BK12. **(a)** Glucose, fructose and sucrose content in pericarp and columella tissues. **(b)** Summed values of these three soluble carbohydrates in pericarp and columella tissues. Values shown are mean ± SE. Different letters indicate statistically significant differences (Tukey's test, $p < 0.05$) among fruit development stages within each genotype. Asterisks indicate statistically significant differences (Student's t-test, $p < 0.05$) between *au* and WT at each fruit development stage.

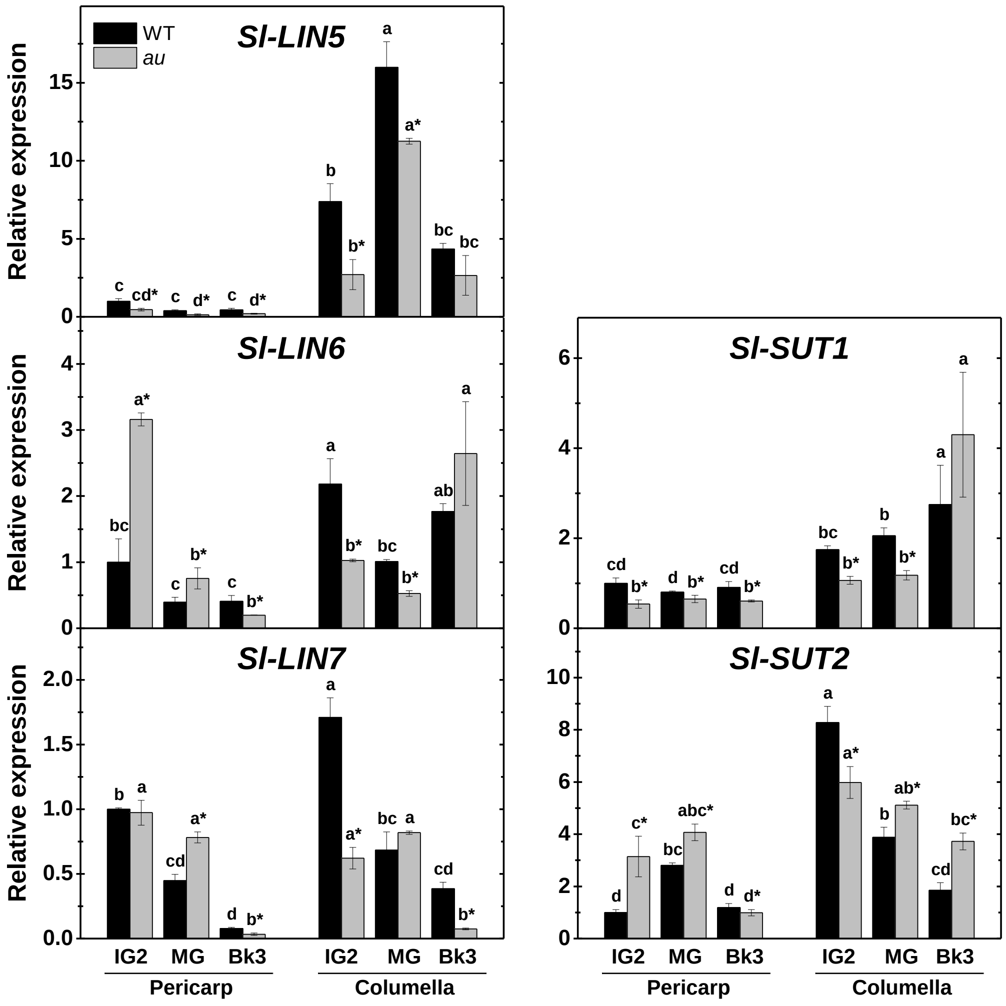

Supplementary Figure 7. Transcript abundance of sink-related genes in developing tomato fruits. Relative mRNA levels of tomato genes encoding invertases (*Sl-LIN*) and sucrose transporters (*Sl-SUT*) in the pericarp and columella tissues of wild-type (WT) and *aurea* (*au*) fruits harvested at immature (IG2), mature green (MG) and ripening (Bk3) stages. Mean relative expression was normalized against pericarp WT samples at IG2 stage. Values shown are mean ± SE. Different letters indicate statistically significant differences (Tukey's test, $p < 0.05$) among fruit development stages within each genotype. Asterisks indicate statistically significant differences (Student’s t-test, $p < 0.05$) between *au* and WT at each fruit development stage.

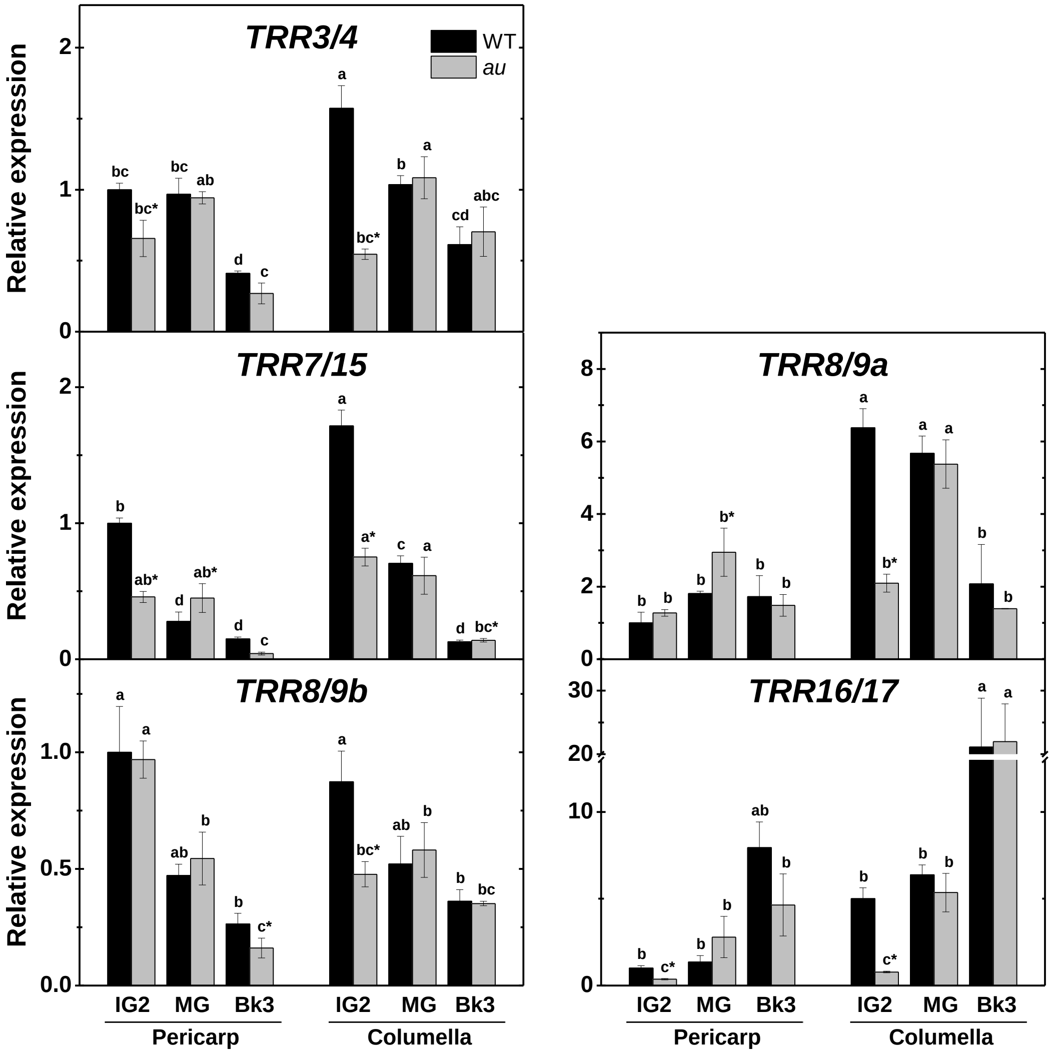

Supplementary Figure 8. Transcript abundance of *TRR* genes in developing tomato fruits. Relative mRNA levels of *TOMATO RESPONSE REGULATOR* (*TRR*) in the pericarp and columella tissues of wild-type (WT) and *aurea* (*au*) fruits harvested at immature (IG2), mature green (MG) and ripening (Bk3) stages. Mean relative expression was normalized against pericarp WT samples at IG2 stage. Values shown are mean ± SE. Different letters indicate statistically significant differences (Tukey's test, $p < 0.05$) among fruit development stages within each genotype. Asterisks indicate statistically significant differences (Student’s t-test, $p < 0.05$) between *au* and WT at each fruit development stage.

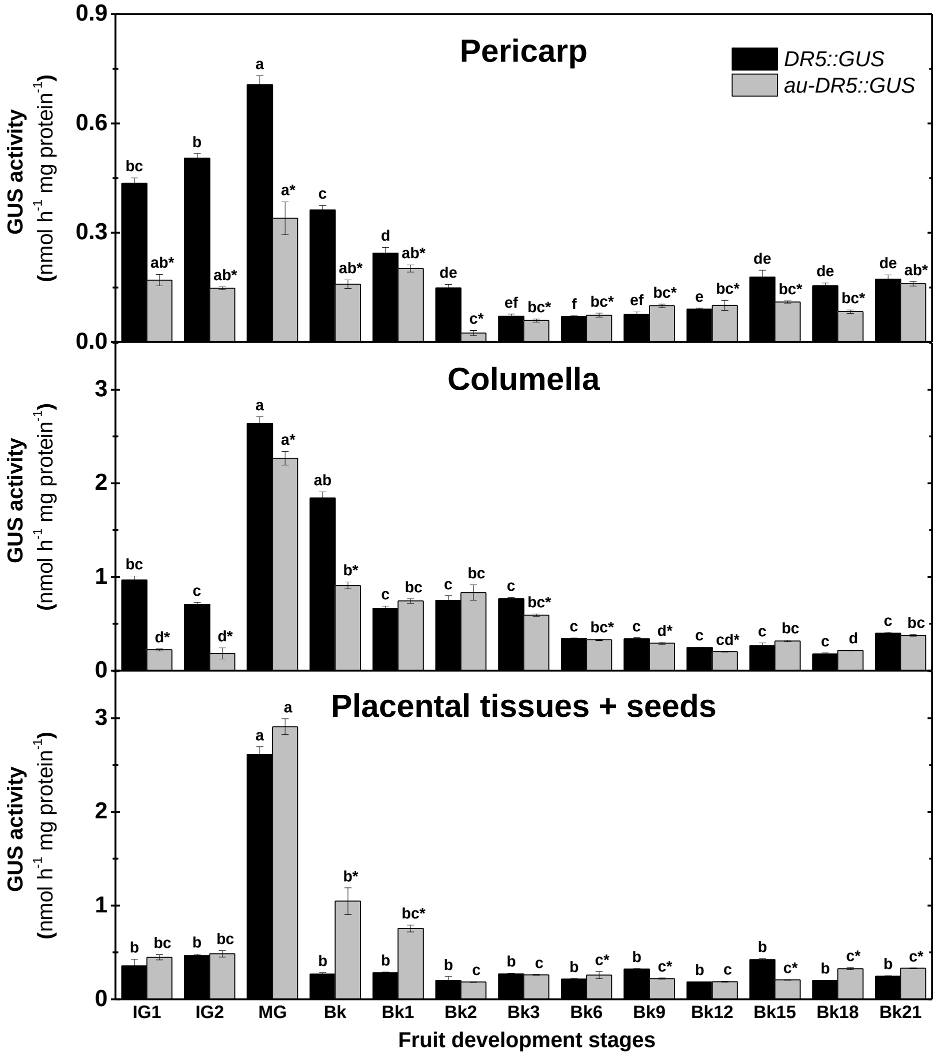

Supplementary Figure 9. Auxin signaling output in developing tomato fruits. *In vitro* GUS activity assayed in the pericarp, columella and placental tissues + seeds of developing wild-type (WT) and *aurea* (*au*) fruits carrying the auxin-responsive promoter *DR5* fused to the GUS reporter protein (*DR5::GUS* and *au-DR5::GUS*). Values shown are mean ± SE. Different letters indicate statistically significant differences (Tukey's test, $p < 0.05$) among fruit development stages within each genotype. Asterisks indicate statistically significant differences (Student's t-test, $p < 0.05$) between *au* and WT at each fruit development stage.

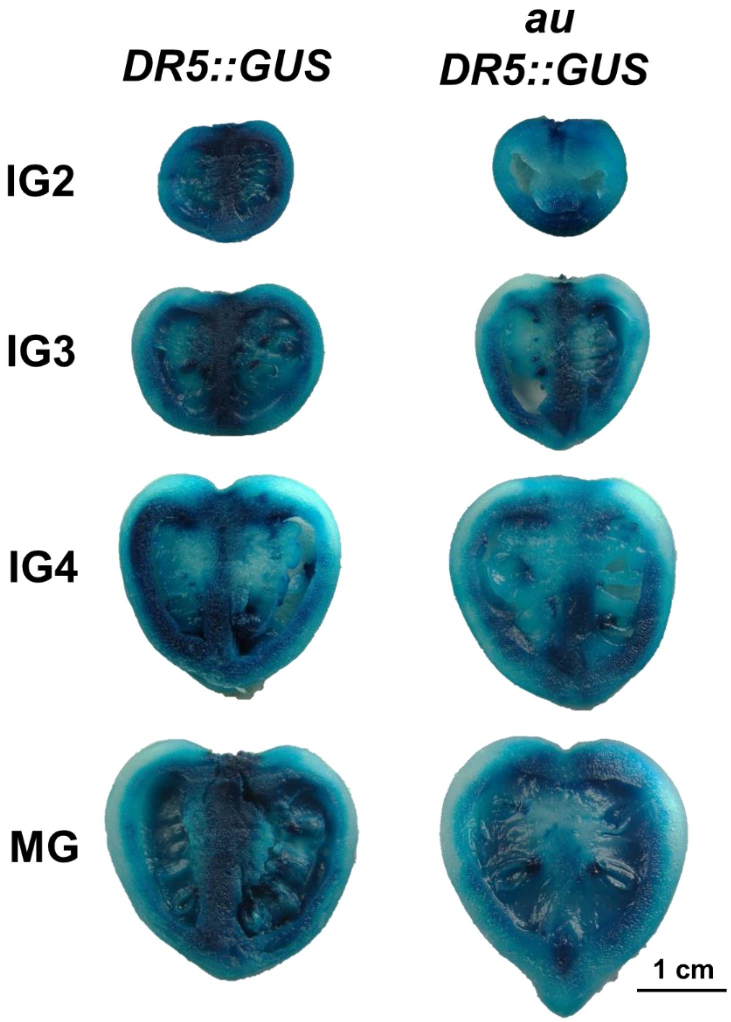

Supplementary Figure 10. Spatial pattern of *DR5::GUS* expression during fruit development. Histochemical GUS staining assayed in immature green fruits (IG2 to IG4) and mature green (MG) fruits of wild-type (WT) and *aurea* (*au*) carrying the auxin-responsive promoter *DR5* fused to the GUS reporter protein (*DR5::GUS* and *au-DR5::GUS*). Bar: 1 cm.

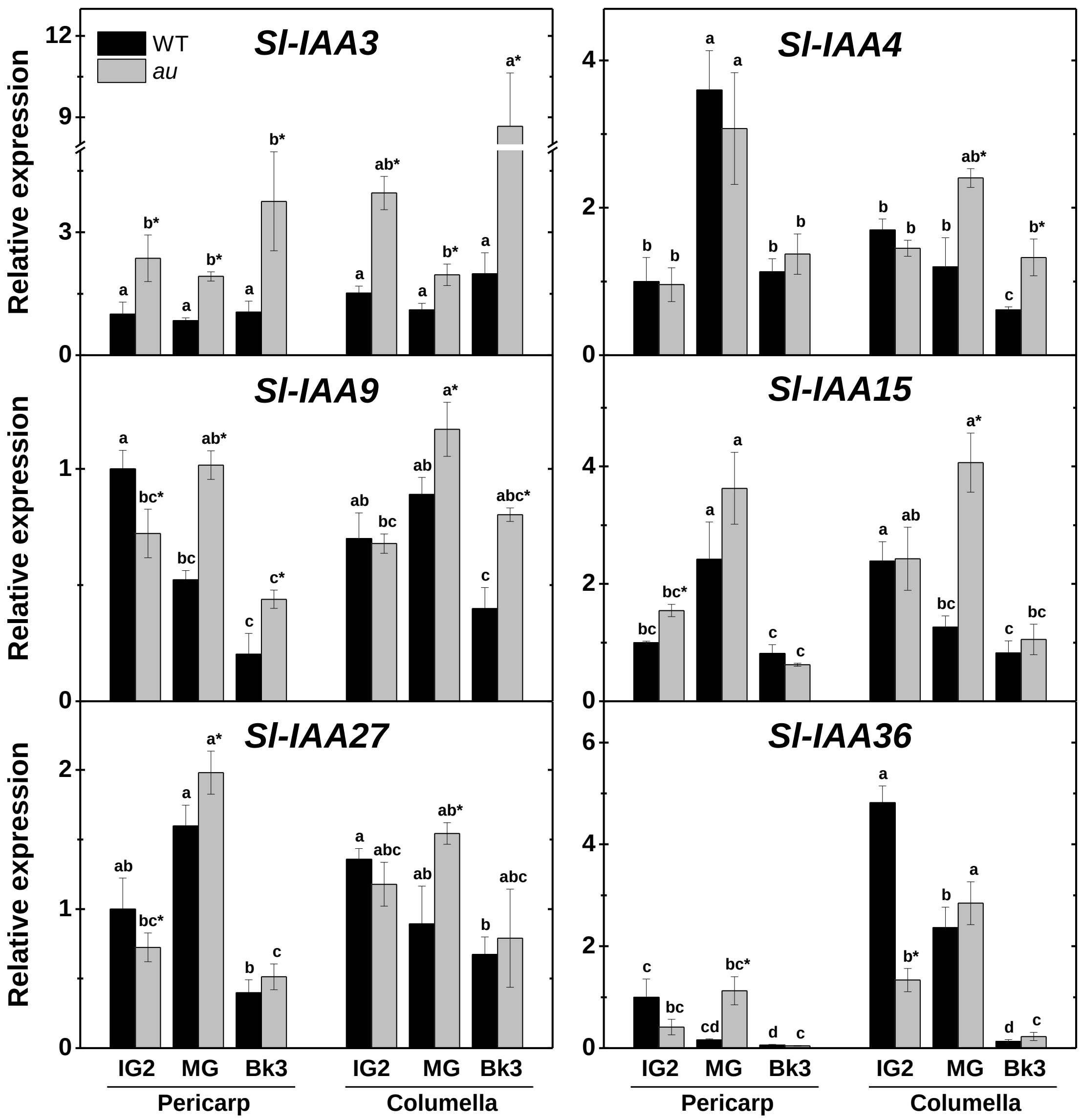

Supplementary Figure 11. Transcript abundance of *Aux/IAA* genes in developing tomato fruits. Relative mRNA levels of the *AUXIN/INDOLE-3-ACETIC ACID (Aux/IAA)* tomato genes *Sl-IAA3, Sl-IAA4, Sl-IAA9, Sl-IAA15, Sl-IAA27* and *Sl-IAA36* in pericarp and columella tissues of wild-type (WT) and *aurea* (*au*) fruits harvested at immature (IG2), mature green (MG) and ripening (Bk3) stages. Mean relative expression was normalized against pericarp WT samples at IG2 stage. Values shown are mean ± SE. Different letters indicate statistically significant differences (Tukey's test, $p < 0.05$) among fruit development stages within each genotype. Asterisks indicate statistically significant differences (Student's t-test, $p < 0.05$) between *au* and WT at each fruit development stage.

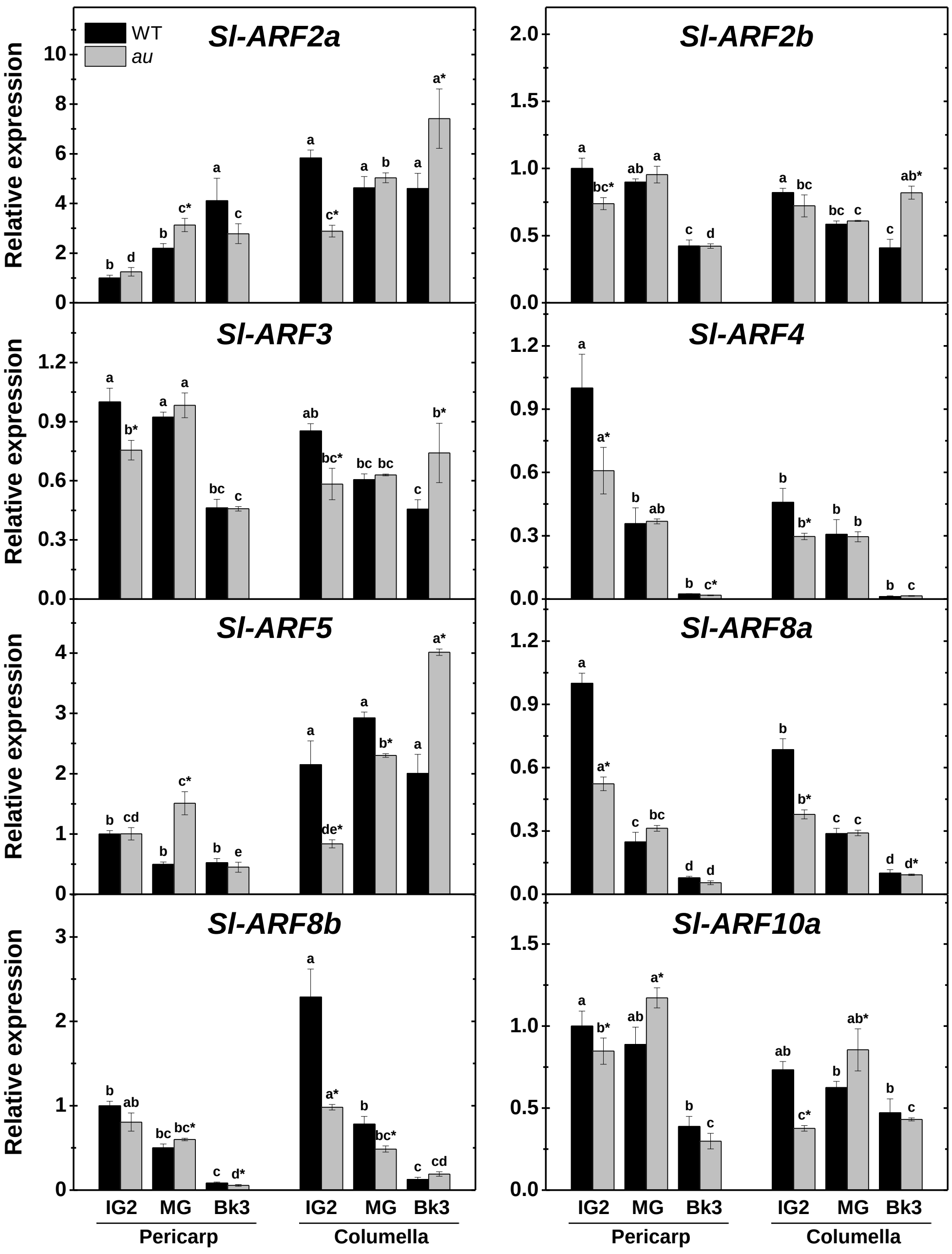

Supplementary Figure 12. Transcript abundance of *ARF* genes in developing tomato fruits. Relative mRNA levels of *AUXIN RESPONSE FACTOR* (*ARF*) tomato genes *Sl-ARF2a*, *Sl-ARF2b*, *Sl-ARF3*, *Sl-ARF4*, *Sl-ARF5*, *Sl-ARF8a*, *Sl-ARF8b* and *Sl-ARF10a* in pericarp and columella tissues of wild-type (WT) and *aurea* (*au*) fruits harvested at immature (IG2), mature green (MG) and ripening (Bk3) stages. Mean relative expression was normalized against pericarp WT samples at IG2 stage. Values shown are mean ± SE. Different letters indicate statistically significant differences (Tukey's test, $p < 0.05$) among fruit development stages within each genotype. Asterisks indicate statistically significant differences (Student's t-test, $p < 0.05$) between *au* and WT at each fruit development stage.

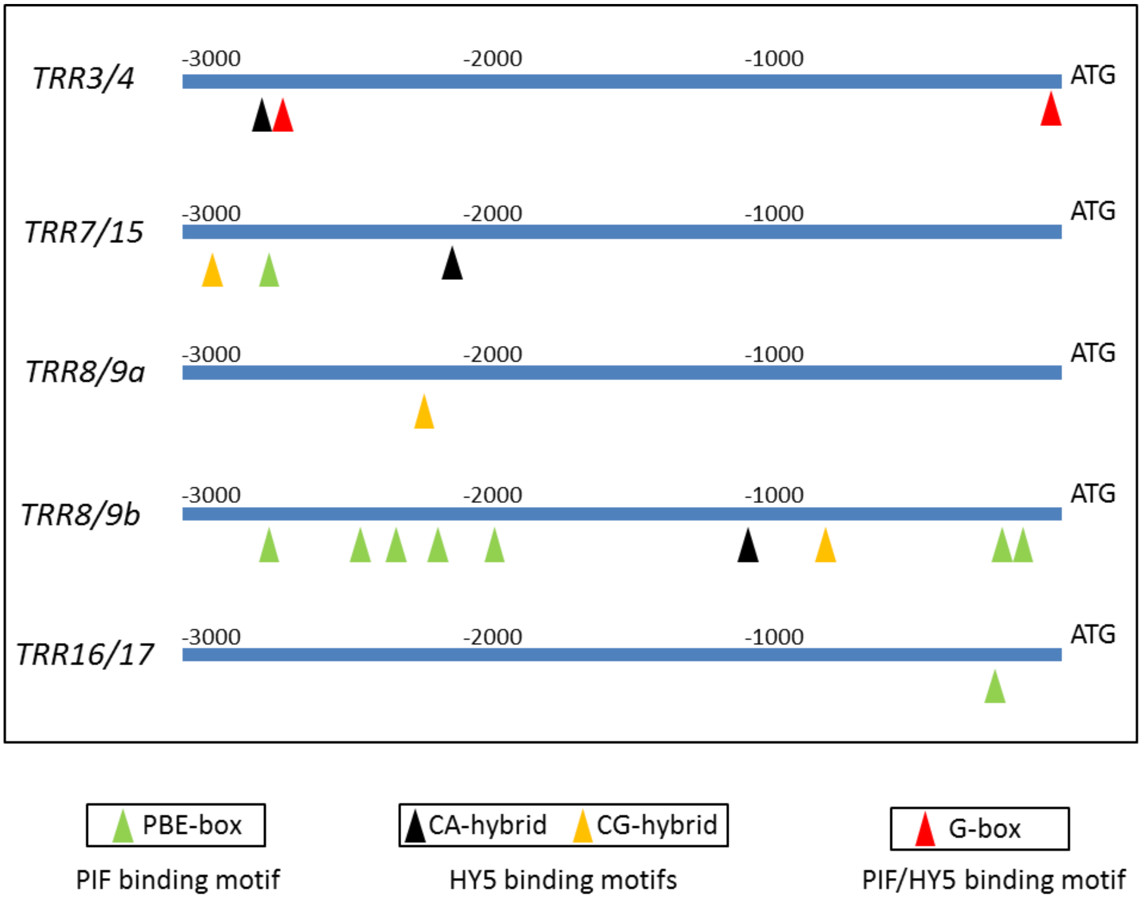

Supplementary Figure 13. HY5- and PIF-binding motifs identified in the promoter region of *TRR* genes. Fragments of 3 kb upstream ATG initiation site of genes encoding type-A TOMATO RESPONSE REGULATORs (TRRs) are represented by a blue line. Motif positions are indicated by triangles: PBE-box (green), recognized by PHYTOCHROME-INTERACTING FACTORs (PIFs). CA hybrid (black) and CG hybrid (yellow), recognized by LONG HYPOCOTYL 5 (HY5). G-box (red), recognized by both PIFs and HY5.

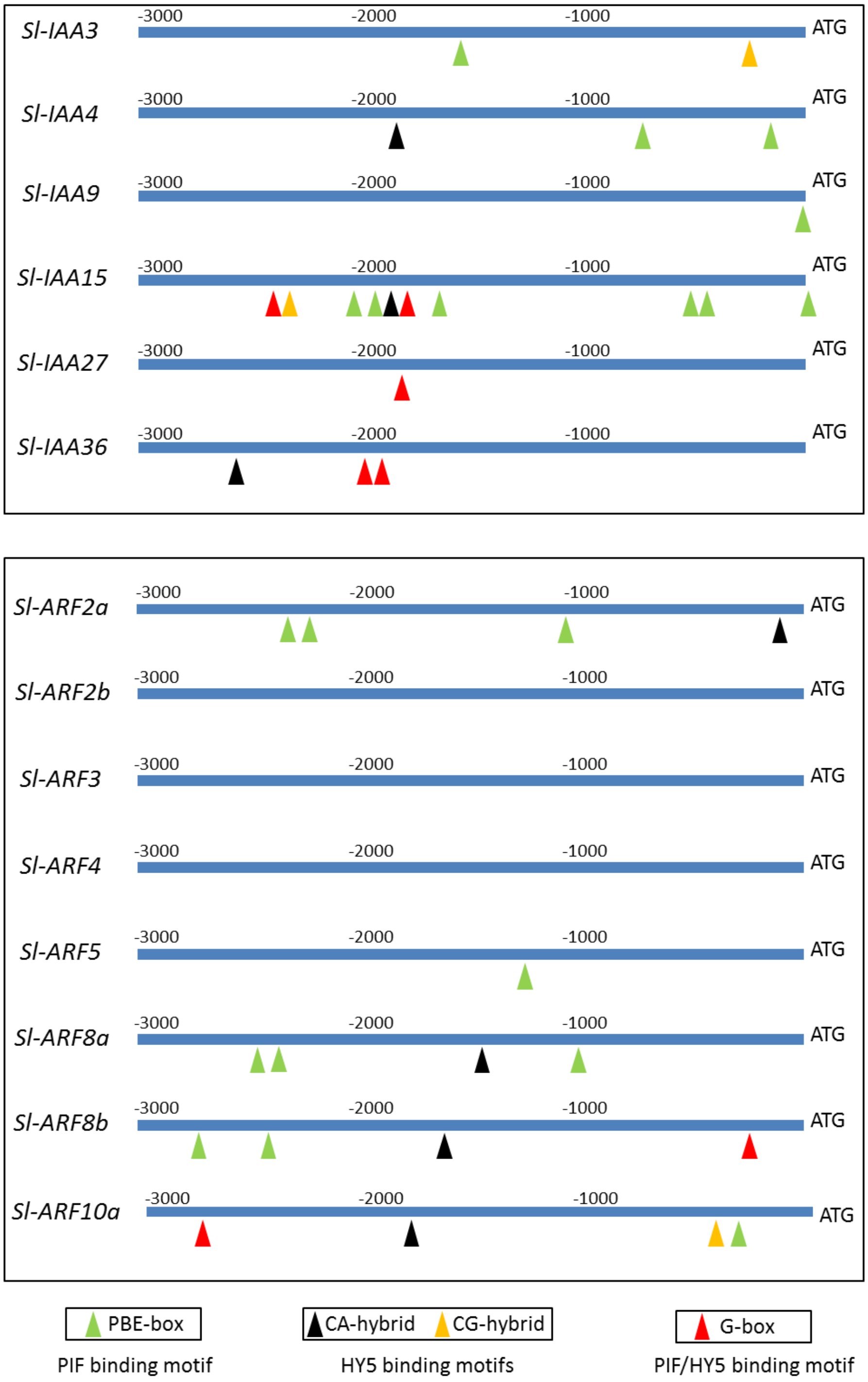

Supplementary Figure 14. HY5- and PIF-binding motifs identified in the promoter region of *Aux/IAA* and *ARF* tomato genes. Fragments of 3 kb upstream ATG initiation site of *AUXIN/INDOLE-3-ACETIC ACID (Aux/IAA) and AUXIN RESPONSE FACTOR* (*ARF*) tomato genes are represented by a blue line. Motif positions are indicated by triangles: PBE-box (green), recognized by PHYTOCHROME-INTERACTING FACTORs (PIFs). CA-hybrid (black) and CG-hybrid (yellow), recognized by LONG HYPOCOTYL 5 (HY5). G-box (red), recognized by both PIFs and HY5.

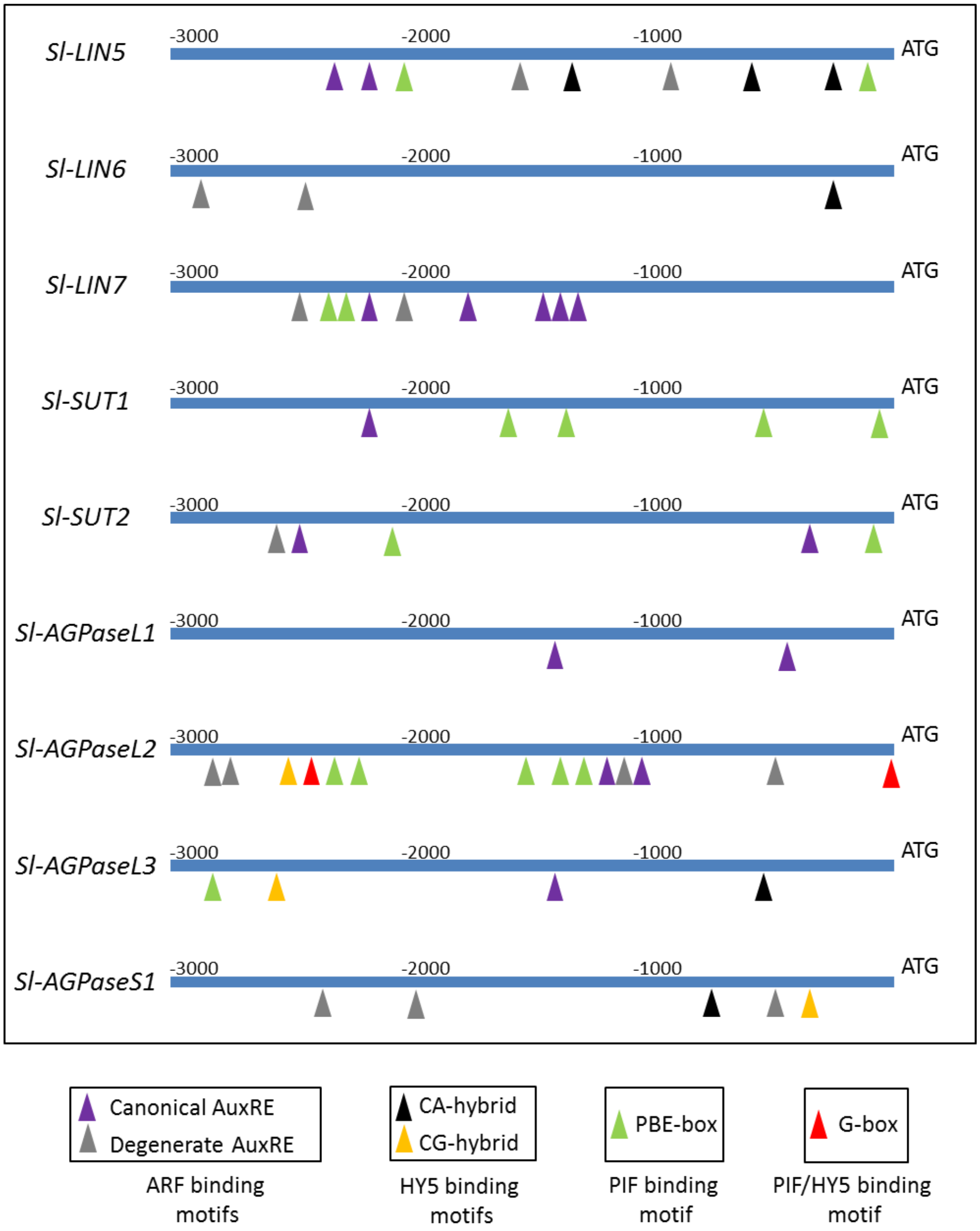

Supplementary Figure 15. HY5-, PIF- and ARF-binding motifs identified in the promoter region of sink- and starch biosynthesis-related tomato genes. Fragments of 3 kb upstream ATG initiation site of the tomato genes encoding cell-wall invertases (*Sl-LIN*), sucrose transporters (*Sl-SUT*) and the small and large subunits of AGPases (*Sl-AGPase*) are represented by a blue line. Motif positions are indicated by triangles: PBE-box (green), recognized by PHYTOCHROME-INTERACTING FACTORs (PIFs). CA-hybrid (black) and CG-hybrid (yellow), recognized by LONG HYPOCOTYL 5 (HY5). G-box (red), recognized by both PIFs and HY5. Canonical AuxRE (purple) and degenerated AuxRE (gray), recognized by AUXIN RESPONSE FACTORs (ARFs).

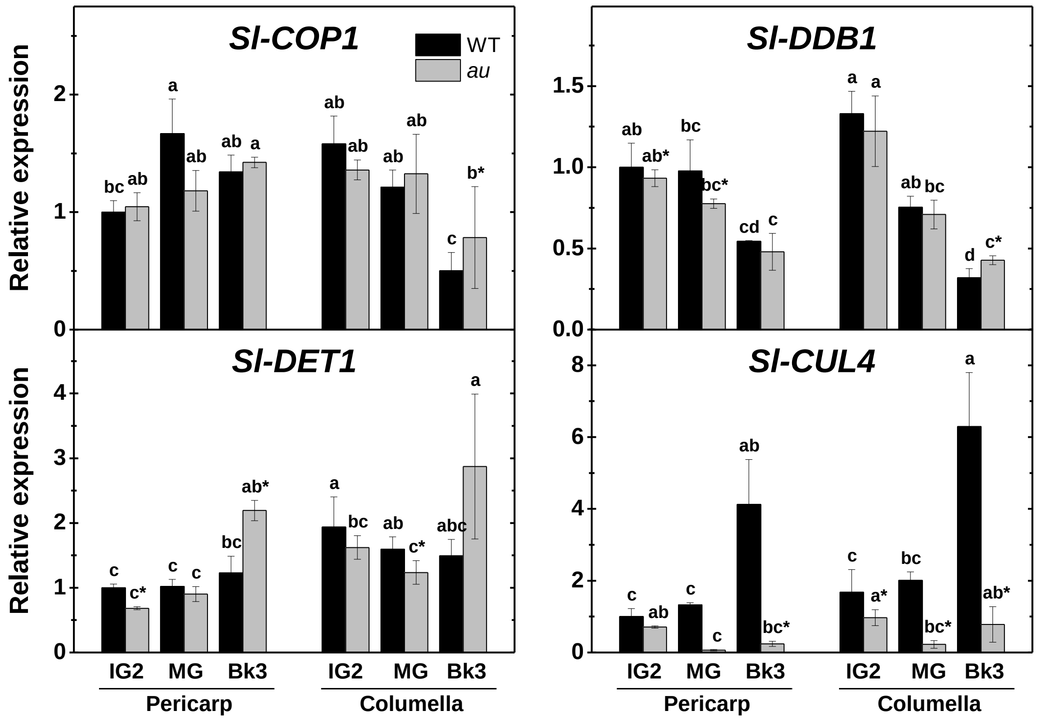

Supplementary Figure 16. Transcript abundance of light signaling-related genes in developing tomato fruits. Relative mRNA levels of tomato genes encoding light signaling associated genes in pericarp and columella tissues of wild-type (WT) and *aurea* (*au*) fruits harvested at immature (IG2), mature green (MG) and ripening (Bk3) stages. Mean relative expression was normalized against pericarp WT samples at IG2 stage. Values shown are mean ± SE. Different letters indicate statistically significant differences (Tukey's test, $p < 0.05$) among fruit development stages within each genotype. Asterisks indicate statistically significant differences (Student's t-test, $p < 0.05$) between *au* and WT at each fruit development stage. The abbreviations indicate the following: *Sl-COP1*, *CONSTITUTIVE PHOTOMORPHOGENIC 1*; *Sl-CUL4*, *CULLIN 4*; *Sl-DET1*, *DE-ETIOLATED1*; *Sl-DDB1*, *UV-DAMAGED DNA BINDING PROTEIN 1*.

Table S1. Relative transcript ratio of *Sl-PHY* genes in pericarp and columella tissues.

		Sl-PHYA	*Sl-PHYB1*	*Sl-PHYB2*	*Sl-PHYE*	*Sl-PHYF*
**IG2 Pericarp**	***Sl-PHYA***	1.0 ± 0.2	3.0 ± 0.6	0.2 ± 0.03	2.0 ± 0.4	0.8 ± 0.1
	Sl-PHYB1	0.3 ± 0.1	1.0 ± 0.4	0.05 ± 0.02	1.2 ± 0.5	0.3 ± 0.1
	Sl-PHYB2	**6.8 ± 2.2**	**20.2 ± 6.4**	**1.0 ± 0.3**	**13.9 ± 4.4**	**5.3 ± 1.6**
	Sl-PHYE	0.5 ± 0.2	1.4 ± 0.6	0.1 ± 0.0	1.0 ± 0.4	0.4 ± 0.2
	Sl-PHYF	1.3 ± 0.46	3.8 ± 1.3	0.2 ± 0.1	2.6 ± 0.9	1.0 ± 0.4
**MG Pericarp**	***Sl-PHYA***	1.0 ± 0.2	0.6 ± 0.13	0.1 ± 0.02	1.6 ± 0.4	0.9 ± 0.1
	Sl-PHYB1	1.4 ± 0.4	1.0 ± 0.28	0.1 ± 0.04	1.1 ± 0.3	0.9 ± 0.2
	Sl-PHYB2	**9.8 ± 1.9**	**7.0 ± 1.3**	**1.0 ± 0.2**	**15.7 ± 3**	**6.4 ± 1.2**
	Sl-PHYE	0.6 ± 0.3	0.5 ± 0.2	0.06 ± 0.03	1.0 ± 0.4	0.4 ± 0.2
	Sl-PHYF	1.5 ± 0.4	1.1 ± 0.3	0.2 ± 0.04	3.8 ± 0.6	1.0 ± 0.2
**Bk3 Pericarp**	***Sl-PHYA***	1.0 ± 0.5	0.5 ± 0.2	0.1 ± 0.06	0.7 ± 0.3	1.6 ± 0.7
	Sl-PHYB1	2.2 ± 0.2	1.0 ± 0.10	0.3 ± 0.03	1.0 ± 0.1	1.0 ± 0.2
	Sl-PHYB2	**7.6 ± 0.8**	**3.6 ± 0.4**	**1.0 ± 0.1**	**5.3 ± 0.6**	**6.9 ± 0.8**
	Sl-PHYE	1.5 ± 0.2	0.7 ± 0.07	0.2 ± 0.02	1.0 ± 0.1	1.3 ± 0.1
	Sl-PHYF	1.1 ± 0.2	0.5 ± 0.08	0.2 ± 0.02	1.0 ± 0.1	1.0 ± 0.2
**IG2 Columella**	***Sl-PHYA***	**1.0 ± 0.3**	**1.2 ± 0.2**	**0.1 ± 0.04**	**8.1 ± 2.2**	**1.0 ± 0.1**
	Sl-PHYB1	0.8 ± 0.2	1.0 ± 0.3	0.1 ± 0.03	1.1 ± 0.3	0.9 ± 0.2
	Sl-PHYB2	7.8 ± 1.5	9.6 ± 1.6	1.0 ± 0.2	63 ± 12.4	9.1 ± 1.5
	Sl-PHYE	0.1 ± 0.04	0.2 ± 0.04	0.02 ± 0.01	1.0 ± 0.32	0.1 ± 0.04
	Sl-PHYF	0.8 ± 0.2	1.0 ± 0.2	0.1 ± 0.02	6.3 ± 1.3	1.0 ± 0.2
**MG Columella**	***Sl-PHYA***	1.0 ± 0.4	0.4 ± 0.2	0.1 ± 0.03	2.5 ± 1.1	0.5 ± 0.1
	Sl-PHYB1	2.6 ± 0.5	1.0 ± 0.2	0.2 ± 0.03	1.1 ± 0.2	1.7 ± 0.3
	Sl-PHYB2	**16.5 ± 4.1**	**6.4 ± 1.6**	**1.0 ± 0.3**	**42 ± 10.7**	**11.0 ± 2.7**
	Sl-PHYE	0.4 ± 0.1	0.2 ± 0.05	0.02 ± 0.01	1.0 ± 0.3	0.3 ± 0.1
	Sl-PHYF	1.5 ± 1.1	0.6 ± 0.4	0.1 ± 0.07	2.4 ± 1.8	1.0 ± 0.3
**Bk3 Columella**	***Sl-PHYA***	1.0 ± 0.5	0.7 ± 0.2	0.4 ± 0.1	2.7 ± 0.8	6.5 ± 0.9
	Sl-PHYB1	3.0 ± 1.0	1.0 ± 0.6	0.9 ± 0.3	1.6 ± 0.8	8.1 ± 2.8
	Sl-PHYB2	**4.8 ± 0.9**	**2.3 ± 0.4**	**1.0 ± 0.5**	**9.1 ± 1.7**	**13.0 ± 2.4**
	Sl-PHYE	0.7 ± 0.3	0.3 ± 0.1	0.2 ± 0.08	1.0 ± 0.5	1.9 ± 0.7
	Sl-PHYF	0.5 ± 0.1	0.3 ± 0.07	0.2 ± 0.05	0.8 ± 0.3	1.0 ± 0.5

Sl-PHYB2 relative expression compared to other *Sl-PHY*s is highlighted in bold terms. Values represent means ± SD from at least three biological replicates and are expressed as relative transcript amount comparing each *Sl-PHY* gene. The abbreviations *Sl-PHYA*, *Sl-PHYB1*, *Sl-PHYB2*, *Sl-PHYE*, *Sl-PHYF* indicate genes encoding tomato PHYTOCHROME A, B1, B2, E and F, respectively. Stages: immature (IG2), mature green (MG) and ripening (Bk3) stages.

Table S2. Primer sequences used for qPCR.

Gene	Primers for qRT-PCR[1]	Locus[2]
Sl-PHYA	F: 5´ - CACTCTCGTGGAGGATTCAT - 3´/ R: 5´ - GAGCCATAAAACACACACCC - 3´	Solyc10g044670
Sl-PHYB1	F: 5´ - ACTTCTGTTCGGTCCATTCC - 3´ / R: 5´ - TCTCAGACAACTGTGATGCC - 3´	Solyc01g059870
Sl-PHYB2	F: 5´ - GTGAGGGTTATTCAGGATGA - 3´ / R: 5´ - TGACCATATACTGAGGGTGAC - 3´	Solyc05g053410
Sl-PHYE	F: 5´ - CGCTATTGAGGAACCCACTT - 3´ / R: 5´ - GCATCAACACCAATCAGACC - 3´	Solyc02g071260
Sl-PHYF	F: 5´ - ACTAGCCAAGATCATTGACG - 3´/ R: 5´ - CTCCAAGATTGAACTCACAAG - 3´	Solyc07g04548
Sl-COP1	F: 5´ -TTGCCCCTGTTGTTCTCA-3´ / R: 5´ - CCAATGAATGACGAAACTGTTC - 3´	Solyc12g005950
Sl-CUL4	F: 5´ - GGAGGAATTGGAGGGGACATTA - 3´ / R: 5´ - GAAATCATGGACTTTTCAGCATCA - 3´	Solyc02g021470
Sl-DDB1	F: 5´ - ATCTCGTGCCTGGACATAAA - 3´ / R: 5´ - CGAAGGAACACATCAGAACA - 3´	Solyc02g021650
Sl-DET1	F: 5´ - GCTATTGACCGCCATAGACA - 3´ / R: 5´ - TCTTAGTTCGCCCATCTGTG - 3´	Solyc01g056340
Sl-LIN5	F: 5´ - TTGGAAGGGATTGAGAATCG - 3´ / R: 5´ - AATTCCAGCCCATCCTTTCT - 3´	Solyc09g010080
Sl-LIN6	F: 5´ - AACCCGCTATCTACCCGTCT - 3´ / R: 5´ - GGGCTTGATCCACTTACGAA - 3´	Solyc10g083290
Sl-LIN7	F: 5´ - TCTTGACTTTGGCTGGGTTC - 3´ R: 5´ - TTCACGACGCACTGAGTTTC - 3´	Solyc09g010090
Sl-SUT1	F: 5´ AGGAGGAAACTTGCCTGGAT - 3´ / R: 5´ - CGACTGCTGGTTTAGCATCA - 3´	Solyc11g017010
Sl-SUT2	F: 5´ - GGAGAGAAGTCTATCACG - 3´ / R: 5´ - GACAAATACAATGAAGTTGC - 3´	Solyc05g007190
Sl-AGPaseL1	F: 5´ - AGCAGACTACTACCAAACAG - 3´ / R: 5´ - ATTCCAATCGGTACTTTCC - 3´	Solyc01g109790
Sl-AGPaseL2	F: 5´ - CTGAAATTATCCCTTCTGCTG- 3´ / R: 5´ - ACTTCACTGTTCCAATATCCTC - 3´	Solyc07g019440
Sl-AGPaseL3	F: 5´ - CGCGCTACTTCGTAATAACC - 3´ / R: 5´ - CCATCAATTCTCCATTGCA - 3´	Solyc01g079790
Sl-AGPaseS1	F: 5´ - TGTAAGATTCACCATTCCGT - 3´ / R: 5´ - TCTTCTATAATTGCTCCCTCTG - 3´	Solyc07g056140
TRR 3/4	F: 5´ - CCAACATCACCAACAAGACG - 3´ / R: 5´ - ATGGGAGAGGAGGGCTTAAA - 3´	Solyc05g006420
TRR 7/15	F: 5´ ATGTTTGGAGGAAGGTGCTG - 3´ / R: 5´TGATTGTGTCGTTGGATCGT - 3´	Solyc03g113720
TRR 8/9a	F: 5´ - TGCTTAGAAGAAGGGGCAGA - 3´ / R: 5´ - GGGGGCTTTTACATTTGGTT - 3´	Solyc10g079600
TRR 8/9b	F: 5'- CAGCAGCAACAACAACAACAG - 3' / R: 5'- TCTGCCCAGCCCTTCTTAGATC -3'	Solyc02g071220
TRR 16/17	F: 5´ - GGTCTAAGGGCGTTGGAGTA - 3´ / R: 5´ - ATCTGAGCCCCTTCTTCCAT - 3´	Solyc06g048930
Sl-IAA3	F: 5´ - GCCACCAGTTCGATCATACA - 3´ / R: 5´ - ATAAGGTGCTCCATCCATGC- 3´	Solyc09g065850
Sl-IAA4	F: 5´ - ACTCCACCTGTTGCCAAGAC - 3´ / R: 5´ - AGATAAGGGGCTCCATCCAT- 3´	Solyc06g053840
Sl-IAA9	F: 5´ - CAGAGGGGAAGTTTCTGTCG - 3´ / R: 5´ - CAACCTGTGCCTTTGTAGCA- 3´	Solyc04g076850
Sl-IAA15	F: 5´ - ATCGGAGACAGCCAAATCAG - 3´ / R: 5´ - TTTGCTGGAGGTTTGTTTCC- 3´	Solyc03g120390
Sl-IAA27	F: 5´ - GCAAGAGAAGCTCAGTGA - 3´ / R: 5´ - ACATCTCCCAAGGAACATCG- 3´	Solyc03g120500
Sl-IAA36	F: 5´ - TGGAAGGAGTAGCCATTGGA - 3´ / R: 5´ - TGGTACATCTCCAGCAAGCA- 3´	Solyc06g066020
Sl-ARF2a	F: 5´ - GCAAGGTCAAGAGTTATCGA - 3´ / R: 5´ - CATTGGTTTCTCAGACAAGTC- 3´	Solyc03g118290
Sl-ARF2b	F: 5´ - CTGGGTTAAGCGACAAGCTC - 3´ / R: 5´ - CCCCGCATTTGATACAGAGT - 3´	Solyc12g042070
Sl-ARF3	F: 5´ - GATTGTTTTGCTCCCTTGGA - 3´ / R: 5´ - GTGGCTGACCCCGATAGATA - 3´	Solyc02g077560
Sl-ARF4	F: 5´ - TGAAAGCCATCAACTCTCGG - 3´ / R: 5´ -ATCCCATCTGACCATCAAGCATC - 3´	Solyc11g069190
SlARF5	F: 5´ - TTCCGAGCCAAGAAAAGAAA - 3´ / R: 5´ - CACTCGCATCAGTTGGAAGA - 3´	Solyc04g081240
SlARF8a	F: 5´ - AGCCCGTCCAATATGTTCAG- 3´ / R: 5´ - TTTGATGGTTGCTTCTGCTG - 3´	Solyc03g031970
SlARF8b	F: 5´ - CATCTCCTTCCGACCACAGT - 3´ / R: 5´ - TGGTGGATCAATTTGTCCTGC- 3´	Solyc02g037530
Sl-ARF10a	F: 5´ - ATTCTCTGTGCCTAGATACTG - 3´ / R: 5´ - CTATAAATGTGCCTAAACTTCCA - 3´	Solyc11g069500
Sl-ERF.E1	F: 5´ -GTTCCTCTCAACCCCAAACG - 3´ / R: 5´ - TTCATCTGCTCACCACCTGTAGA- 3´	Solyc09g075420
Sl-ERF.E2	F: 5´ - ACTTCGTGAGGAAACCCTGAAC - 3´ / R: 5´ - GTTACTAATATAAGTCATGTTGGG - 3´	Solyc06g063070
Sl-ERF.E4	F: 5´ - AGGCCAAGGAAGAACAAGTACAGA - 3´ / R: 5´ - CCAAGCCAAACGCGTACAC - 3´	Solyc01g065980
CAC (constitutive)	F:5´ - CCTCCGTTGTGATGTAACTGG - 3´ / R:5 -ATTGGTGGAAAGTAACATCATCG - 3´	Solyc08g006960
Expressed (constitutive)	F: 5´ - GCTAAGAACGCTGGACCTAATG - 3´ / R: 5´ - TGGGTGTGCCTTTCTGAATG - 3´	Solyc07g025390

[1]F: forward, R: reverse

[2]Locus according to the Sol Genomics Network database (http://solgenomics.net/).
